# Exploring the Mitogenomes of Mantodea: New Insights from Structural Diversity and Higher-Level Phylogenomic Analyses

**DOI:** 10.3390/ijms241310570

**Published:** 2023-06-24

**Authors:** Qinpeng Liu, Yingqi Liu, Qiaoqiao Liu, Li Tian, Hu Li, Fan Song, Wanzhi Cai

**Affiliations:** Department of Entomology and MOA Key Lab of Pest Monitoring and Green Management, College of Plant Protection, China Agricultural University, Beijing 100193, China; sznbmail@sina.com (Q.L.); yingqiliu0720@163.com (Y.L.); 2323@cau.edu.cn (Q.L.); ltian@cau.edu.cn (L.T.); tigerleecau@hotmail.com (H.L.)

**Keywords:** Mantodea, mitochondrial genome, gene rearrangements, phylogenomics

## Abstract

The recently reorganized classification of Mantodea has made significant progress in resolving past homoplasy problems, although some relationships among higher taxa remain uncertain. In the present study, we utilized newly sequenced mitogenomes and nuclear gene sequences of 23 mantid species, along with published data of 53 mantises, to perform familial-sampling structural comparisons of mantodean mitogenomes and phylogenomic studies. Our rstructural analysis revealed generally conserved mitogenome organizations, with a few cases of tRNA gene rearrangements, including the detection of *trnL_2_* duplication for the first time. In our phylogenetic analysis, we found a high degree of compositional heterogeneity and lineage-specific evolutionary rates among mantodean mitogenomes, which frequently corresponded to several unexpected groupings in the topologies under site-homogeneous models. In contrast, the topologies obtained using the site-heterogeneous mixture model fit the currently accepted phylogeny of Mantodea better. Topology tests and four-cluster likelihood mapping analyses further determined the preferred topologies. Our phylogenetic results confirm the monophyly of superfamilial groups Schizomantodea, Amerimantodea, Heteromantodea, Promantidea, and Mantidea and recover the early-branching relationships as (Mantoidoidea + (Amerimantodea + (Metallyticoidea + Cernomantodea))). Additionally, the results suggest that the long-unresolved phylogenetic position of Majangidae should be placed within Mantidea, close to Mantoidea, rather than within Epaphroditoidea. Our findings contribute to understanding the compositional and structural diversity in mantodean mitogenomes, underscore the importance of evolutionary model selection in phylogenomic studies, and provide new insights into the high-level phylogeny of Mantodea.

## 1. Introduction

**Figure 1:**
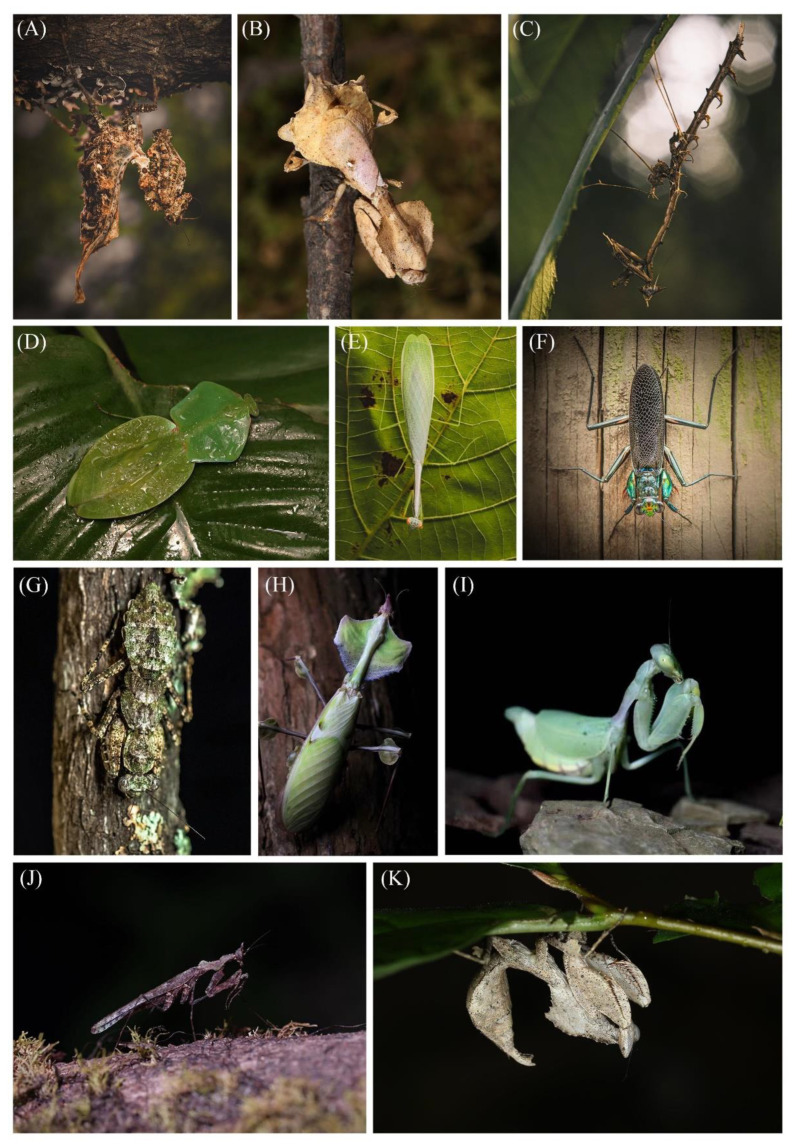
Live habitus images of species representing ten mantodean superfamilies and their polymorphic cryptic strategies: *Pseudacanthops* sp. (Acanthopoidea) (**A**); *Epaphrodita musarum* (Epaphroditoidea: Epaphroditidae) (**B**); *Toxodera denticulata* (Eremiaphiloidea) (**C**); *Choeradodis rhombicollis* (Mantoidea) (**D**); *Leptomantella lactea* (Nanomantoidea) (**E**); *Metallyticus splendidus* (Metallyticoidea) (**F**); *Mintis septemspina* (Gonypetoidea) (**G**); *Idolomantis diabolica* (Hymenopoidea) (**H**); *Cilnia humeralis* (Miomantoidea) (**I**); *Haania hainanensis* (Haanioidea) (**J**); *Brancsikia freyi* (Epaphroditoidea: Majangidae) (**K**). (**A**,**C**,**E**–**G**) by Zeyi Lyu; (**B**,**K**) by Zhaoyang Chen; (**D**,**H**,**I**) by Qinpeng Liu; (**J**) by Mingyuan Fan. All photos are published with permission.

Mantises (Dictyoptera: Mantodea) are well-known predatory insects with more than 2500 recognized species [1]. The most recent classification reorganized the extant Mantodea into 16 superfamilies and 29 families [2], in which the neotropical superfamily Chaeteessoidea is generally accepted as a sister group to all the remaining extant Mantodea (infraorder Spinomantodea). These insects have a global distribution, and many species are known for their polymorphic cryptic strategies or shared highly homoplastic characteristics among lineages [3] (Figure 1). Thus, mantises are considered an ideal model for exploring adaptive phenotypic and convergent evolution.

Although the monophyly of Mantodea and its sister group’s relationship with Blattodea has been generally accepted [4,5,6], many phylogenetic relationships within Mantodea remain unresolved [2,7]. Historical interpretations of phylogenetic relationships relying on morphological characteristics alone can be unnatural, as completely unrelated lineages occupying similar habitats can display convergent adaptive traits [7,8,9].

In the past two decades, research on mantodean phylogeny has been facilitated by integrating molecular, morphological, and fossil data. Grimaldi [10] provided a cladistic analysis of early-branching relationships within Mantodea using the morphological characteristics of fossil and extant species, which is largely concordant with the currently accepted framework (Figure S1A). Subsequent tentative analysis for resolving mantodean deep-relationships is mostly conducted with the integration of nuclear and mitochondrial loci. Svenson and Whiting [8] attempted to resolve the phylogeny of 55 mantodean species using five gene markers (Figure S1B). Their study revealed discordance between morphological-based classification and molecular phylogenetic trees. Svenson and Whiting [9] further confirmed the massive unnatural grouping of higher taxa in mantodean systems using nine genes from 288 mantodean samples representing 27 families in 15 superfamilies (Figure S1D), which indicated that mantodean systematics is in dire need of revision. A comprehensive morphological cladistic analysis conducted by Wieland [3] confirmed that the majority of mantis morphological characteristics are indeed ecomorphic convergences rather than examples of homology. This topology was later proven to be incongruent with phylogenies based on molecular, genital, or chromosomal data, along with several historical systematic arrangements [2,11,12,13]. A recent systematic phylogenetic study of Mantodea by Svenson and Rodrigues [14] included the endemic Caribbean family Epaphroditidae for the first time (Figure S1G) and elucidated a remarkable biogeographic history of this family in the Greater Antilles. However, relationships within Mantodea have not been widely discussed, and relationships among some major clades and the monophyly of several superfamilies remain unclear (Figure S1). To date, only reports on relationships among certain higher taxa have reached general agreement. For instance, relationships among early branches (Chaeteessoidea + (Mantoidoidea + Schizomantodea)) are generally well-supported by fossil, morphological, and molecular data [1,2,9,10,15]. The sister relationship of Amerimantodea and Cernomantodea as well as the systematics of Amerimantodea have also been well-established by a comprehensive study of the auditory system by Yager and Svenson [16], molecular phylogenetic analyses by Svenson and Whiting [9] and Svenson and Rodrigues [14], and morphological studies by Rivera and Svenson [7]. In Cernomantodea, the monophyly of Heteromantodea, Promantidea, and Mantidea (Hymenopoidea + Mantoidea clade) is supported by independent studies with some terminal relationships that have been properly resolved [2,9,14,16,17].

The use of mitochondrial genome (mitogenome) sequences to resolve deep intra- and inter-ordinal relationships of insects show considerable promise for higher taxon coverage, overcoming missing data, and yielding more acceptable computation loads, thus facilitating multiple comparisons among datasets and evolutionary models [18,19,20,21]. However, mitogenomic research on Mantodea has largely focused on comparative genomics covering limited taxa [22,23,24,25,26,27,28]. In a recent mitochondrial phylogenomic study by Ma et al. [29], species from 10 superfamilies and 15 families were sampled, but several early-branching lineages of Cernomantodea and extensive Amerimantodea species, as well as several families with the evolutionary significance of Mantodea, have not been included in mitochondrial phylogenomic studies to date. Moreover, although mitochondrial markers serve as the dominant signal contributors in previous phylogenetic studies of mantises [9], results from the current mitochondrial phylogenomics are generally not approved by systematists for recovering unexpected and conflicting relationships.

Lineage-specific compositional heterogeneity, variable rates of substitution in sequences, and sparse taxon sampling have been proven to contribute to systematic errors in phylogenetic analyses [18,30,31,32]. Model adequacy has been confirmed to be critical for ensuring accurate tree reconstruction in molecular phylogenomic studies [20]. The site-heterogeneous CAT-based model implemented in PhyloBayes [33] has been shown both theoretically and empirically to be effective in suppressing the attraction artifacts [34,35,36]. However, in Mantodea, previous attempts to reconstruct intra-ordinal relationships using molecular markers all used site-homogeneous models that did not account for variation in composition or exchange rates across the data.

In the present study, we aimed to explore the mitochondrial gene rearrangements of the order Mantodea and produce a relatively robust backbone of the mantodean phylogeny using larger molecular datasets. We sequenced the mitogenomes of 23 species and three nuclear genes [*histone 3* (*H3*), *18S* rRNA, and *28S* rRNA] sequences of 21 species of Mantodea. Combined with published data, the phylogenomic study covered all families of Mantodea excluding Chaeteessidae. Majangidae was first included in the phylogenetic analysis; 14 families (Mantoididae, Angelidae, Coptopterygidae, Acanthopoidae, Liturgusidae, Chroicopteridae, Majangidae, Epaphroditidae, Hoplocoryphidae, Miomantidae, Galinthiadidae, Rivetinidae, Empusidae, and Dactylopterygidae) and six superfamilies (Mantoidoidea, Chroicopteroidea, Epaphroditoidea, Hoplocoryphoidea, Miomantoidea, and Galinthiadoidea) were studied using a mitogenomic approach for the first time. Our finding suggested that multiple tRNA duplications are prevalent across the whole Spinomantodea. Among the Mantodea, the Eremiaphiloidea superfamily had the highest abundance of gene rearrangements, with the first detection of *trnL_2_* duplication. In phylogenetic analysis, we specifically employed the site-heterogeneous CAT + GTR model to suppress systematic errors in site-homogeneous models caused by compositional heterogeneity. Topology tests and four-cluster likelihood mapping (FcLM) analyses were also conducted to determine the preferred topologies, which addressed the higher-level phylogeny of Mantodea with a particular focus on several lineages whose affiliation or monophyly remains uncertain.

## 2. Results

### 2.1. Mantodean Mitogenome Structure

**Figure 2:**
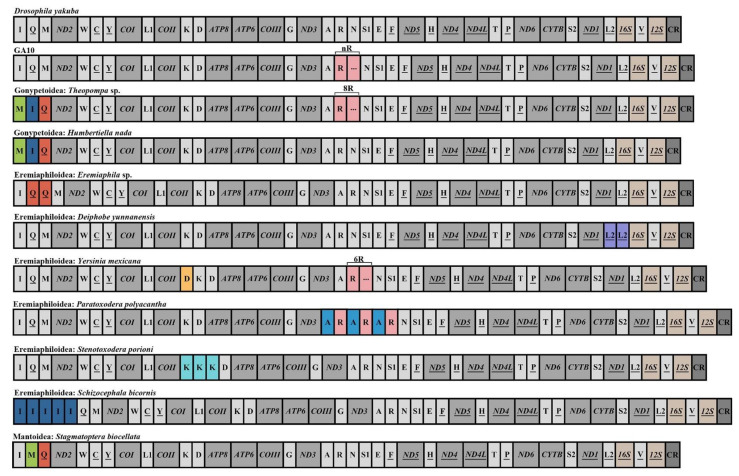
Mitochondrial gene rearrangements of the 76 species in Mantodea. tRNA genes are indicated by their one-letter corresponding amino acids, and tRNA gene rearrangements are indicated in color. Abbreviations: *ATP6* and *ATP8*, ATP synthase subunits 6 and 8, respectively; *COI*–*COIII*, cytochrome oxidase subunits 1–3; *CYTB*, cytochrome b; *ND1*–*6* and *ND4L*, NADH dehydrogenase subunits 1–6 and 4L, respectively; *16S* and *12S*, large and small rRNA subunits, respectively; CR, control region. The underlined label indicates the gene was transcribed from the minority strand. GA10, gene arrangements of ten mantodean species (n = 2: *Angela* sp., *Euchomenella heteroptera*, *Choeradodis rhombicollis*, *Hymenopus coronatus*, *Theopropus elegans*, *Mantis religiosa*, *Statilia maculata*; n = 3: *Orthodera ministralis*; n = 4: *Thesprotiella* sp., *Hestiasula* sp.).

We obtained the complete mitogenomes of 23 newly sequenced species, with lengths ranging from 15,499 bp in *Epaphrodita musarum* to 16,186 bp in *Thesprotiella* sp. Most species contained 37 genes (two rRNAs, 13 PCGs, and 22 tRNAs) and a large non-coding region, except for *Deiphobe yunnanensis*, *Eremiaphila* sp., *Euchomenella heteropteran*, *Choeradodis rhombicollis*, *Thesprotiella* sp., and *Angela* sp., for which several tRNA duplications were observed (Figure 2). Annotation revealed two identical *trnL_2_* copies with a 73 bp spacer inserting between them in *Deiphobe yunnanensis* and two identical *trnQ* copies with a 51 bp spacer between them in *Eremiaphila* sp. Multiple duplications of *trnR* were commonly found in two derived species, *Euchomenella heteropteran* and *Choeradodis rhombicollis*, as well as in two amerimantodean species, *Angela* sp. and *Thesprotiella* sp. Combined with the published sequences, three rearrangement hotspots were observed among the 76 mantodean mitogenomes, viz. tRNA gene clusters *CR*-*I-Q-M*-*ND2*, *COX2*-*K-D*-*ATP8*, and *ND3*-*A*-*R*-*N*-*S*-*E*-*F*-*ND5*, while the duplication of *trnL_2_* in the *ND1-L_2_-16S* gene cluster was first recorded in Mantodea (Figure 2).

### 2.2. Nucleotide Composition and Evolutionary Rates of Mantodean Mitogenomes

The heterogeneity of sequence variation was assessed using AliGROOVE separately for different datasets (Figure S2). In general, the results revealed higher heterogeneity in Empusidae, *Metallyticus splendidus*, *Thesprotiella* sp., *Haania* sp., and *Macromantis hyalina* than in the remaining lineages. A significantly accelerated evolution was found in the third codon positions of mantodean mitochondrial PCGs (mean similarity score = 0.139), with mainly negative similarity scores in many early-branching superfamilies. Decreased sequence heterogeneity was found in the datasets with the removal of the third codon positions (Figure S2). In the analyses of compositional diversity, sequences of all species revealed high A + T content values ranging between 69.2–77.4%. Among the 15 superfamilies, Haanioidea exhibited the highest A + T content (76.4 ± 0.8%). The contrasting compositions of nucleotides were also found between two subfamilies of Gonypetoidea, Gonypetinae and Iridopteryginae, which presented A + T content of 70.1 ± 1.6% and 74.8 ± 0.4%, respectively. Such intra-lineage divergences may result in the distant placement of related species and produce false groupings of other unrelated taxa exhibiting similar base compositions. The evolutionary rates evaluated by Ka/Ks values of the 76 mantodean mitogenomes ranged between 0.15–0.28 and also differed greatly among families and superfamilies (*p* < 0.001, ANOVA). An accelerated evolutionary rate was observed for Haanioidea (Ka/Ks ratio = 0.25). These results indicate lineage-specific evolutionary rates among the mantodean mitogenomes. Because of such notably higher compositional divergence, Haanioidea species may not be robustly placed or may be misplaced in topologies.

### 2.3. Phylogenetic Analyses under Site-Homogenous Models and Parsimony Methods

**Figure 3:**
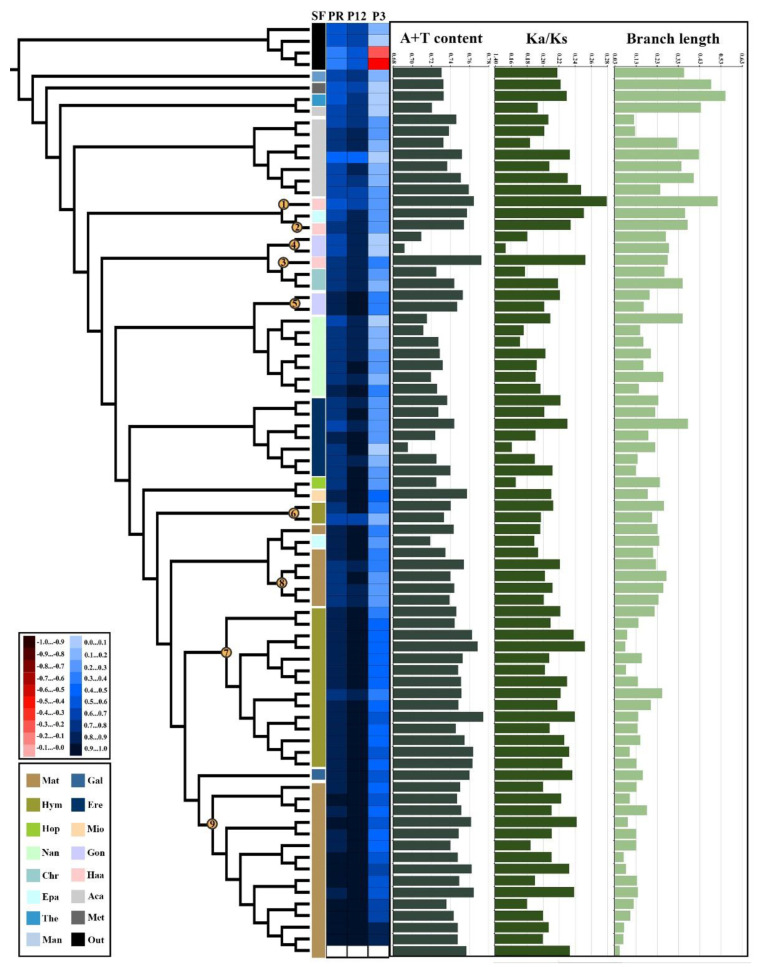
Systematic errors in phylogenetic analyses under site-homogeneous models. The rooted ML tree obtained from PCGRNA-no partition analysis was chosen to plot mean similarity scores among sequences, A + T content, Ka/Ks, and branch length values. Numbers on the topology (1–9) indicate the identifier of the corresponding clades or terminals: (1) *Haania* sp., (2) *Caliris* sp., (3) *Arria pallida*, (4) Gonypetinae, (5) Iridopteryginae, (6) Empusidae, (7) Hymenopodidae, (8) Deroplatyidae, (9) Mantidae. The mean similarity score among the sequences is represented by colored squares (upper legend) based on AliGROOVE scores ranging from −1, indicating a great difference in rates from the remainder of the dataset (red coloring), to +1, indicating rates matching all other comparisons (blue coloring). The A + T contents (%) and branch lengths were derived from the PCGRNA dataset while the Ka/Ks values were calculated based on 13 PCGs. Abbreviation: SF: superfamily; PR: PCGRNA dataset; P12: PCG12 dataset; P3: PCG3 dataset; Mat, Mantoidea; Gal, Galinthiadoidea; Hym, Hymenopoidea; Ere, Eremiaphiloidea; Hop, Hoplocoryphoidea; Mio, Miomantoidea; Nan, Nanomantoidea; GManon, Gonypetoidea; Chr, Chroicopteroidea; Haa, Haanioidea; Epa, Epaphroditoidea; Aca, Acanthopoidea; The, Thespoidea; Met, Metallyticoidea; Man, Mantoidoidea; Out, Outgroups.

Maximum likelihood analyses of the four datasets and three data-optimized strategies under site-homogenous models consistently presented monophyletic results of the following groups (Table 1; Figures S3–S14): the superfamily groups Heteromantodea and Promantidea (bootstrap value (BP) > 0.95) and the superfamilies Chroicopteroidea, Nanomantoidea, and Eremiaphiloidea. However, the phylogenetic relationships of other lineages were incongruent across different datasets and partition schemes. The well-established infraorder Schizomantodea was not recovered as monophyletic in the PCGRNA/NGPCGRNA-gene partition analyses (Figures S4 and S10). In Artimantodea, Amerimantodea failed to be recovered as a monophyletic group in all analyses, and Mantidea was recovered as monophyletic only in the NGPCGRNA-gene partition but with a low BP (0.49) (Figure S10). In the early-branched superfamilies, the positions of Metallyticoidea were highly variable, including the sister group to all remaining Mantodea and the cluster with Thespoidea or Angelidae (Figures S5, S7, S8, S11, and S13), which were likely to be false groupings because the three groups shared very similar base compositions and evolutionary rates (Figure 3). In the early branches of Cernomantodea, Haanioidea may serve as the rogue lineage, with variable grouping with Thespoidea, Epaphroditidae, Chroicopteroidea, or Gonypetinae across the twelve analyses, rendering these topologies unstable in phylogeny. In the more derived superfamily Cernomantodea, Empusidae tended to be placed outside the remaining Promantidea in the analyses with the third codon positions reserved (Figures S3, S5, and S11), rendering Hymenopoidea paraphyletic. Galinthiadoidea was nested within the (Mantoidea + Hymenopoidea) clade in all analyses, except the NGPCGRNA-gene partition. These results are inconsistent with the currently accepted relationships within Mantodea. In conclusion, ML analyses using site-homogeneous models presented limited potential and identified very unstable relationships across different datasets and partitioning schemes, especially with regard to early branches.

Maximum parsimony analyses resulted in less resolved and supported trees overall, compared to the model-based phylogeny (Table 1; Figures S15–S18), which may reflect the heterogeneous sequence divergence of mantodean mitogenomes among lineages to some extent. Nevertheless, the monophyly of the superfamily group Schizomantodea and superfamilies Nanomantoidea, Eremiaphiloidea, and Chroicopteroidea was recovered in all MP analyses.

### 2.4. Systematic Errors under Site-Homogeneous Models

We performed error analyses in mitochondrial phylogenomics by mapping the results of A + T content, Ka/Ks value, and branch length on the phylogenetic tree obtained from PCGRNA-no partition analysis under site-homogenous models (Figure 3). An overall decreasing tendency of sequence heterogeneity and branch length as well as high divergence of A + T content and Ka/Ks ratio were observed.

For AliGROOVE analyses, several lineage-specific divergences of mantodean mitogenomes were identified, which corresponded to several unexpected groupings of these lineages under ML topologies (Figure 3 and Figures S2–S14). For example, in Hymenopoidea, although all sequences presented positive similarity scores, a highly heterogeneous sequence divergence was found between Hymenopodidae and Empusidae with mean similarity scores of 0.76 and 0.61, respectively (Figure 3, clades 6 and 7). Lineage-specific divergences were also found between Caliridinae and Haaniinae in Haanioidea (Figure 3, clades 1, 2, and 3), Gonypetinae and Iridopteryginae in Gonypetoidea (Figure 3, clades 4 and 5), and Deroplatyidae and Mantidae in Mantoidea (Figure 3, clades 8 and 9). These taxa presented intra-lineage heterogeneous sequence divergence and were prone to be placed distantly in ML topologies.

On a larger scale, some lineages were more heterogeneous in the intra-lineage nucleotide composition than others, and their inner relationships under the site-homogeneous models were also relatively sensitive to model or dataset changes. For example, the early-branching Cernomantodea (including Chroicopteroidea, Haanioidea, Gonypetoidea, and Nanomantoidea) showed an average A + T content value of 73.3 ± 4.0%. In contrast, Promantidea presented a more homogeneous variation in nucleotide composition with an average A + T content value of 75.0 ± 1.2%. The relationships among the four earlier branching superfamilies also varied under ML topologies. On a smaller scale, some false groupings of the unrelated taxa were likely caused by compositional convergence. For example, the unexpected grouping of Metallyticoidea and Thespoidea, which were recovered frequently under different ML analyses, showed very close composition and evolutionary rates in the sequences. In addition, some related species with different A + T contents, such as Gonypetinae (70.1 ± 1.6%) and Iridopteryginae (74.8 ± 0.4%) in Gonypetoidea, were consistently placed distantly in ML phylogenies, further confirming the results of the heterogeneous analyses above. The contrasting Ka/Ks values among the lineages also demonstrated the heterogeneity of the mantodean mitogenome. Species that presented a high A + T content generally had a notable Ka/Ks value (e.g., haanioidean lineages). Overall, these results revealed a high degree of heterogeneous sequence divergence in mantodean mitogenomes among lineages, which may severely impede the phylogenetic reconstruction using site-homogeneous models.

### 2.5. Phylogenetic Analyses under Site-Heterogeneous Model

**Figure 4:**
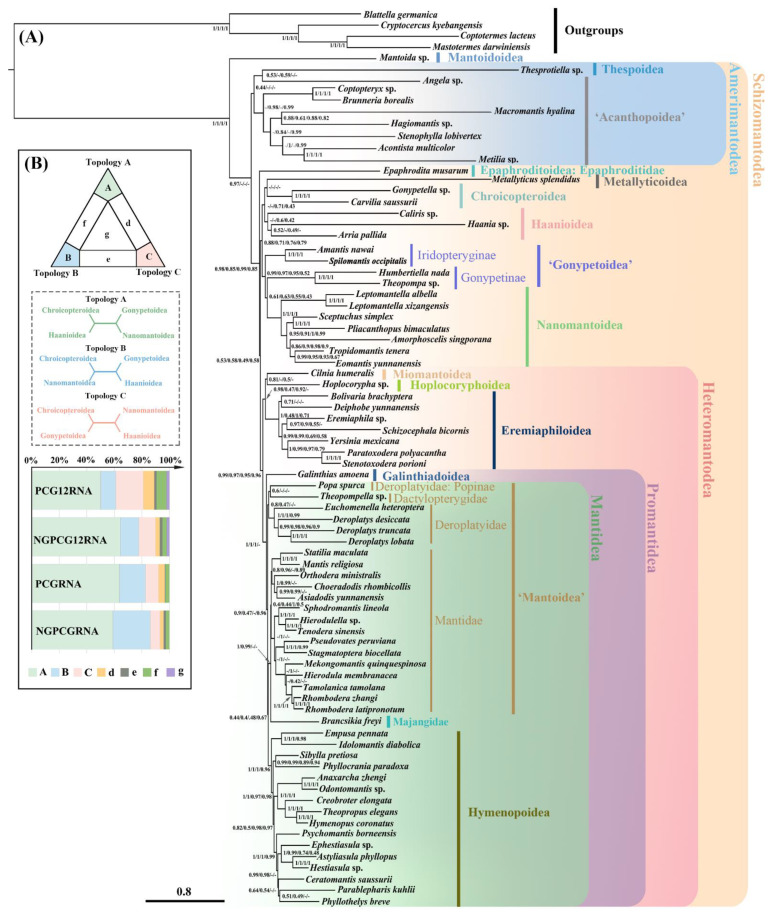
Phylogeny of Mantodea inferred from PhyloBayes analyses of four datasets under the site-heterogeneous mixture CAT + GTR model (**A**). Supports at nodes are Bayesian posterior probabilities; the order from left to right is NGPCGRNA, PCGRNA, NGPCG12RNA, and PCG12RNA. BPPs lower than 0.4 are indicated as ‘−’. The phylogenetic contents support the current topologies of the four datasets with respect to the four unstable superfamilies (Haanioidea, Gonypetoidea, Nanomantoidea, and Chroicopteroidea) using FcLM (**B**).

Bayesian analyses of the four datasets under the CAT + GTR model produced topologies with relatively stable relationships that were more congruent with the currently accepted phylogenetic results (Figure 4A; Table 1; Figures S19–S22). For early-branching clades, the monophyly of the infraorder Schizomantodea was recovered in all analyses. The monophyly of Amerimantodea was recovered using the NGPCGRNA dataset. The monophyly of the majority of superfamilies was also recovered with the exception of Acanthopoidea, Gonypetoidea, and Mantoidea. For superfamilial relationships, Mantoidoidea was strongly supported as the sister group of the remaining mantodean lineages [Bayesian posterior probabilities (BPP) = 1], thus supporting the hypothesis (Mantoidoidea + Schizomantodea). The unexpected grouping of Metallyticoidea and other superfamiles (e.g., as a sister group with Thespoidea), which was supported by several site-homogeneous model analyses, was no longer recovered. Schizomantodea was recovered as (Amerimantodea + (Epaphroditoidea + (Metallyticoidea + remaining Cernomantodea))), in which the (Metallyticoidea + remaining Cernomantodea) clade was further divided into two main lineages. One of the deep branches was ((Metallyticoidea + Haanioidea + Chroicopteroidea) + (‘Gonypetoidea’ + Nanomantoidea)), although the monophyly of Gonypetoidea was not supported. Two subfamilies of Gonypetidae in Gonypetoidea were frequently placed distantly in our ML analyses, as well as in existing mitochondrial phylogenomics [24,28,37], while they were consistently found to be paraphyletic and grouped together with Nanomantoidea under the CAT + GTR model. Another branch, Heteromantodea, clustered Miomantoidea, Hoplocoryphoidea, and Eremiaphiloidea together as a sister group of Promantidea with BPP greater than 0.90. In addition, Galinthiadoidea was convincingly placed outside Mantidea in analyses using PCGRNA, NGPCGRNA, and NGPCG12RNA datasets (BPP = 1) (Figures S19–S21), indicating a more distant relationship with Hymenopoidea. This finding was congruent with previous morphological and molecular discussions (Figure S1C–I). The unexpected polyphyly of Hymenopoidea, which frequently occurred under homogeneous models from PCGRNA and NGPCGRNA datasets, was not recovered, and all Bayesian analyses strongly supported the monophyletic Hymenopoidea (BPP = 1). The unresolved family Majangidae was found to be more derived than what was previously reported and was consistently placed within Mantidea in all analyses as a sister group to Mantidae. The major inconsistencies among topologies, produced with the four datasets using PhyloBayes, were the recovery of Amerimantodea as well as the positions of Metallyticoidea, Haanioidea, and Chroicopteroidea at the base of Cernomantodea. In general, the unexpected relationships of many lineages in homogeneous-based topologies mentioned above that might be attributed to the systematic errors were not observed under the CAT + GRT model in the PhyloBayes analyses, suggesting the “correction” effect of the heterogeneous evolutionary processes in the site-heterogeneous mixture mode.

### 2.6. Testing Alternative Hypotheses

After analyzing the heterogeneous sequence divergence among mantodean mitogenomes, we first employed different data-optimized strategies in the ML analyses to establish the preferred topology. However, as presented above, data partitioning, heterogeneous codon position removal, model selection, and nuclear gene addition under site-homogeneous models showed limited potential to reduce systematic biases. Employing these strategies under the site-homogeneous models also tended to shift the topologies toward the CAT + GTR-based results. Moreover, the CAT + GTR-based topologies showed advantages in higher nodal supports of most recovery clades (Table 1). These results demonstrate that the topologies under the site-heterogeneous model were preferred over those under the site-homogeneous model in the reconstruction of the phylogeny of Mantodea. In addition, previous studies have shown that the topologies obtained under the CAT + GTR mixture model are not defensible under a GTR-based test [38]. Hence, if a test includes both the GTR-based topologies and CAT + GTR-based topologies, the latter will often be strongly rejected. If this situation occurs, further comparison among the PhyloBayes topologies will be unrealistic. Considering these two factors, we did not include ML trees in the subsequent topology analyses.

We performed a topology test under the GTR + G model based on the four datasets to investigate the acceptance of four topologies derived from PhyloBayes and two prior hypotheses at the family level before and after nuclear gene supplementation (Table 2). The two prior hypotheses represent the most recent multi-source molecular loci results [14] and the total evidence taxonomic framework [2]. The former analysis showed that the topology of the NGPCGRNA dataset was more acceptable and better supported by three datasets (NGPCG12RNA, NGPCGRNA, and PCGRNA). This better-supported topology from NGPCGRNA recovered the monophyly of Amerimantodea, which was difficult to establish in other analyses. The latter analysis showed that the taxonomic framework of Schwarz and Roy [2] was strongly supported by all datasets, whereas the hypothesis of Svenson and Rodrigues [14] was only accepted in mitogenome datasets with the addition of nuclear gene fragments (NGPCGRNA and NGPCG12RNA). After combining the two results (Table 2) and the nodal supports in phylogeny (Table 1), we concluded that the supplementation of the nuclear genes improved the topologies in both theoretical and practical analyses. The preferred topologies were thus determined as trees from the NGPCGRNA and NGPCG12RNA datasets under the CAT + GTR model.

The four PhyloBayes topologies consistently recovered the early-branching cernomantodean groups as ((Chroicopteroidea + Haanioidea+ Metallyticoidea) + (‘Gonypetoidea’ + Nanomantoidea)). Aside from Metellyticoidea (see Discussion), the relationships among the remaining four superfamilies were quite variable among the different datasets as well as among the data-optimized strategies in ML analyses. Thus, we employed FcLM to test the alternative relationships of this clade and assess incongruent or confounding signals in each dataset (Figure 4B). The average non-informative proportion of this clade in the four datasets was only 10.9%, indicating that all datasets provided credible information for phylogenetic reconstruction [39]. The results congruently identified the highest support for the relationship ((Chroicopteroidea + Haanioidea) + (Gonypetoidea + Nanomantoidea)) and thus matched the PhyloBayes topologies (Figure 4 and Figures S19–S22).

## 3. Discussion

### 3.1. Gene Arrangements in Mantodean Mitogenomes

The gene arrangement in the majority of Mantodea mitogenomes is conserved and ancestral (Figure 2), with only a small percentage showing rearrangements. The emergence of novel gene rearrangements within this order is primarily attributed to tRNA duplications, inversions, and translocations, with duplications being relatively common [28,39]. Among these gene rearrangement events, the duplication of *trnR* within the *ND3-A-R-N-S-E-F-ND5* region is the most prevalent. Previous studies have identified a few cases of *trnR* duplication in cernomantodean lineages, including Gonypetoidea, Haanioidea, Mantoidea, as well as some Eremiaphiloidea and Hymenopoidea [22,25,28,40,41]. Our study identified two additional cases in Amerimantodea, represented by Angela sp. in Acanthopoidea and Thesprotiella sp. in Thespoidea, indicating that such events occur across the lineages of Mantodea. However, given that most mitochondrial gene arrangements in the aforementioned lineages still conform to ancestral states, we infer that the origin and evolution of multiple *trnR* duplications are likely independent, at least at the level of superfamilies within Mantodea.

Among all superfamilies in the order Mantodea, Eremiaphiloidea appears to have the most diverse range of tRNA rearrangements, with all three known tRNA rearrangement hotspots occurring within this superfamily (Figure 2). Our study identified a novel rearrangement region (*ND1-L_2_-16S*) within this superfamily in *Deiphobe yunnanensis*, bringing the total number of rearrangement regions in Mantodea to four. It is noteworthy that lineages of this superfamily exhibit strong dispersal ability and significant morphological heterogeneity. It is likely that rich tRNA rearrangements accompany the rapid diversification process in this group.

Hereby, although a few cases of tRNA rearrangements have been revealed across mantodean lineages, it is challenging to generalize certain character as a synapomorphy for a family or superfamily. To elucidate the evolution of the gene arrangements in mantodean mitogenomes, future studies require more comprehensive sampling in terms of both fauna and phylogenetic representative in order to avoid the influences of sampling bias, and to be conducted based on robust phylogenetic backgrounds.

### 3.2. Phylogenetic Implication

Our results are the first to show lineage-specific compositional heterogeneity and accelerated evolutionary rates within mantodean mitogenomes. The preliminary steps in phylogenetic reconstruction under site-homogeneous models revealed unsatisfactory results, which were incongruent with the currently accepted taxonomic framework in many aspects. The frequently identified correlation between the false grouping in topologies and nucleotide features may suggest the negative impact of heterogeneity in mantodean phylogenomic analyses, which explained the inability of site-homogeneous models to resolve the higher-level mantodean phylogeny. The use of the site-heterogeneous CAT + GTR model largely overcame the errors of the heterogeneous evolutionary processes mentioned above and provided more stable topologies with better support in cladistic recovery. The results under the site-heterogeneous model (Figure 4) were generally congruent with the current phylogeny of Mantodea (Figure S1C–E,G–I) in several crucial aspects: retrieving Mantoidoidea as the sister group of Schizomantodea, recovering a monophyletic Amerimantodea comprising Acanthopoidea and Thespoidea; supporting Heteromantodea as monophyly including Miomantoidea, Hoplocoryphoidea, Eremiaphiloidea, Galinthiadoidea, Mantoidea, and Hymenopoidea; and recovering the monophyly of the derived mantodean superfamilial group Promantidea and Mantidea. Our study also adds to arguments for the status of Galinthiadoidea to be distant from Hymenopoidea and as the sister of the entire Mantidea. Some key or controversial clades are discussed below.

In the early branching of Cernomantodea, our results consistently recovered a relationship of (Chroicopteroidea + Haanioidea + Metallyticoidea) + (‘Gonypetoidea’ + Nanomantoidea), uniting the five superfamilies as the sister group to Heteromantodea. Although with the unexpected nesting of Metallyticoidea within this clade, the remaining relationships of the four lineages may still provide referable information for systematics. Previous studies have repeatedly shown that the four superfamilies are very unstable among mantodean phylogenies, presented by uncertain relationships among themselves and their subunits (Figure S1B–I). The topology of Svenson and Whiting [9] supported the uniting of Chroicopteroidea with Nanomantoidea as Nanomantodea, but with weak nodal support, and this combination lacks clear morphological synapomorphies [2]. In contrast, our study supported closer relationships of Gonypetoidea with Nanomantoidea and Chroicopteroidea with Haanioidea, corroborating the recent comprehensive sampling research by Svenson and Rodrigues [14]. The retention of the proximal lobe (bl) on the ventral phallomere in these four superfamilies is similar to many early-diverging lineages (e.g., Acanthopoidea), which may support their grouping at the basal split. In addition, many Chroicopteroidea and Haanioidea share a similar wing polymorphism between the sexes, and Gonypetoidea and Nanomantoidea share a weakly sclerotized phallomere complex. The members of Iridopteryginae (subfamily of Gonypetoidea) and Nanomantidae had also been classified together [12,13] for several synapomorphies. The cluster of the four superfamilies was further confirmed with FcLM analyses (Figure 4B), although Gonypetoidea was recovered as paraphyly with respect to Nanomantoidea. Consistent patterns have been identified in other phylogenetic analyses that specifically encompassed oriental lineages within the two superfamilies [26,29]. These recurring patterns are likely attributed to the limited representation of species within the two superfamilies. As a result, it is imperative for future studies to prioritize the sampling of Palearctic and Afrotropic gonypetoids, along with Australasian and Afrotropic nanomantoids.

In Heteromantodea, most of our results support further splitting of the clade as (Promantidea + (Eremiaphiloidea + Miomantoidea + Hoplocoryphoidea)) (Table 1). Although previous phylogenies have shown a sister relationship between Hoplocoryphoidea and Eremiaphiloidea [8,17,42,43] or Hoplocoryphoidea and Miomantoidea [14,16], the unity of all three superfamilies in one clade was recovered in the present study. In Ma et al. [29], although the superfamily Hoplocoryphoidea was not sampled, the sister relationship between Miomantoidea and Eremiaphiloidea was also recovered with strong support in both ML and BI analyses. From a morphological perspective, the females of many species among the three superfamilies have mesopterous to apterous forms. Among Mantidea, the monophyly of Mantoidea has been difficult to recover for variable positions of Dactylopterygidae and Deroplatyidae relative to Mantidae, as indicated in previous studies [14,16,29,42,43]. Our results recovered the topology ((Deroplatyinae + (Popinae + Dactylopterygidae)) + (Majangidae + remaining Mantidea)), which clustered the two Afrotropical lineages together and left the Asian deroplatyids alongside, better fitting the biogeographic distribution. Considering the existing hypotheses of these groups reviewed by Schwarz and Roy [2], we inferred that the placement of the three families, as well as the status of Deroplatyinae and Popinae, should be a focus of future revisionary efforts.

The superfamily Metallyticoidea includes only one genus and five species and has been morphologically well-defined [16,44]. However, the position of Metallyticoidea is still a controversial but important aspect of phylogenetic studies. The problem involves determining early-branching relationships and shaping the current geographic distribution pattern of Mantodea—especially the original continental distribution of Eumantodea [9] and the reservation of Artimantodea [1,3]. Several hypotheses have been proposed in relation to the position of Metallyticoidea, as reviewed by Wieland [3], and two of them are most acceptable to date, viz. the sisters to Artimantodea or nested within Artimantodea. Morphological features and cladistic analysis [2,3,45] are prone to place this superfamily outside the Artimantodea, while molecular or integrated phylogenies with denser species sampling and evolutionary modeling give this group a position nested within Artimantodea [1,9,14,15,16,46,47]. Our results are consistent with the majority of molecular results mentioned above, placing this superfamily within the Artimantodea. However, the nesting of Metallyticoidea in the (Chroicopteroidea + Haanioidea) clade interrupted the monophyly of Cernomantodea in our study and did not explain the evolution of the auditory system parsimoniously [16]. It should also be noted that our study included only a single species of Metallyticoidea, which likely facilitated problematic grouping with the unrelated taxa. Therefore, the relationships among the Metallyticoidea and basal Cernomantodea groups reported here are yet to be validated in future studies.

An interesting finding was the position of the Caribbean lineage Epaphroditidae in the Epaphroditoidea. This family has a long and complicated taxonomic history, with its genera having been placed within Hymenopodinae and Acanthopidae [48], while their phylogenetic position has not been explored until recently. To date, only two studies have included this family in molecular phylogenetic analyses. Svenson et al. [17] first studied this family in a hymenopoidean phylogeny using total evidence (7514 nucleic acid sites and 124 morphological characteristics), placing this group alongside the Eremiaphiloidea. However, this placement was not supported by morphological synapomorphies [2]. Svenson and Rodrigues [14] positioned Epaphroditidae outside the (Caliridinae + Heteromantodea) clade in ML analysis or as a sister to Caliridinae in BI analysis, but both analyses recovered a distant relationship between Caliridinae and Haaniinae in Haanioidea, which may be due to systematic error under the site-homogeneous model. Our analyses under the site-homogeneous model also showed that Epaphroditidae was prone to clustering with Haanioidea lineages, but all these phylogenies placed this family in an outer position of Cernomantodea compared to the above studies. The PhyloBayes analyses consistently recovered this family as a sister to (Cernomantodea + Metallyticoidea) clade, implicating a transitional position from the “Old World” lineages to Neotropical lineages. It is worth noting that the genus *Callimantis* in Epaphroditidae has been shown cytologically to be very close to many Amerimantodea species in low chromosome number and achiasmatic meiosis [2,49].

The endemic family Majangidae from Madagascar was first included in the phylogenetic analysis, represented by the genus *Brancsikia* in Brancsikiinae. Similar to Epaphroditidae [48], the genera of Majangidae have been placed distantly in the classification for their divergent ecomorphs, which include the dead-leaf mimic genus *Brancsikia*, stick mimic genus *Danuriella*, and the bark-dwelling genera *Majanga* and *Liturgusella*. The genus *Brancsikia* included in our study was historically placed within the subfamily Creobotrinae in Mantidae [50] or Deroplatyinae in Deroplatyidae [11,13,51], and was indicated to be closer to Hymenopodidae in the study by Schwarz and Helmkampf [52]. This genus was moved to Epaphroditidae by Roy and Schutte [53] and then removed from their concept of the family based on comparative morphology by Rodrigues and Svenson [48], leaving *Brancsikia incertae sedis*. The recent system placed Epaphroditidae and Majangidae within Epaphroditoidea, mainly based on the morphology of *Epaphrodita* and *Brancsikia* [2]. In all our analyses, although both families were placed as members of Cernomantodea, they were far from each other. Epaphroditidae was placed as the early-branching lineage of Cernomantodea, whereas Majangidae was placed within Promantidea, with more consistent results in all PhyloBayes topologies that recovered *Brancsikia* as the sister group with Mantidae. Evidence that could imply a distant relationship between *Brancsikia* and *Epaphrodita* has been reviewed in detail by Rodrigues and Svenson [48], and our results provide further support. In addition to the extreme morphological similarity that was thought to indicate convergent evolution among *Deroplatys* and *Brancsikia* in previous studies, maternal care behavior, which is often found in tropical mantidean lineages, particularly deroplatyids [54], was also significant in *Brancsikia*. The ootheca in *Brancsikia* and *Deroplatys* are notably similar in terms of external morphology and internal structure [53]. The high degree of sexual dimorphism in body size in many Mantidea species is also present in *Brancsikia*, and the well-developed median secondary distal process (sdpm) and the hook-like apical process of the left phallomere (paa) in most Majangidae also support its close relationship with Mantidae. However, given the contentious status of Deroplatyidae, we cannot directly conclude that Majangidae should be included in Mantoidea. Nevertheless, we can infer the taxonomic position of the family within Mantidea in congruence with early classifications [13,51]. We hypothesized that the morphological similarity and diversity of the two endemic island lineages, Majangidae and Epaphroditidae, are a case of community-level convergence [55,56].

Based on the aforementioned discussion, our phylogenetic study is built upon a representative sampling of higher-level taxa, establishing a robust foundation of data and methodology for future research in mantodean mitochondrial phylogenomics, divergence time estimation, and biogeographical investigations. However, as mentioned above, our current research faces limitations due to the absence of key extant lower-level taxa, such as Gonypetoidea from Palearctic and Afrotropic regions, Nanomantoidea from Australasia and Afrotropic region, as well as species of Chaeteessoidea, which hampers further downstream analyses. The study of mantodean divergence times has also been constrained by various factors. Firstly, the scarcity of fossil records, particularly for recently diverged clades, severely limits the availability of fossils suitable for ingroup calibration [57]. Secondly, there is a lack of research that integrates morphological data from fossil species and extant species using rigorous cladistic analyses, primarily due to the extensive morphological convergence within Mantodea, which poses challenges in character selection. Nevertheless, the study by Demers-Potvin et al. [45] presents an auspicious starting point for the addressed issue. Additionally, comparative research on divergence time estimation in Mantodea, utilizing different fossil selection schemes and prior settings, is still lacking. Recently, Ma et al. [29] estimated the divergence times of Mantodea by adjusting calibration nodes using crown Dictyoptera and other Hexapoda. However, for ingroup calibration, this study exclusively employed *Pseudomantoida extendidera* [58] as a minimum age constraint for the stem of Mantodoidea, while the overreliance on outgroup fossil information may introduce bias due to lineage-specific differences in evolutionary rates [59]. Furthermore, the study imposed strict minimum age constraints and relaxed maximum age constraints on all calibration points, potentially resulting in an overestimation of divergence times for multiple nodes [60]. Notably, the timing of the crown group of Mantodea presents an illustrative example: Svenson and Whiting [9] reported a mean time lag of 70 million years between the stem group and the crown group of Eumantodea; in contrast, in Ma et al. [29], due to the absence of Chaeteessoidea, although the study arrived at a similar time lag, it essentially represents the age from the stem group to the crown group of Spinomantodea. Furthermore, compared to previous studies, Ma et al. [29] also exhibited an overall tendency to overestimate divergence times in several recently diverged lineages of Mantodea [9,14]. Therefore, we conclude that future investigations into the phylogeny and divergence times of Mantodea require the utilization of expanded taxonomic sampling within controversial clades, and the integration of data from extant species and fossil species. Only by employing a more reliable phylogenetic framework and improved calibration priors can we obtain more trustworthy conclusions regarding the mantodean tree of life.

## 4. Materials and Methods

### 4.1. Taxon Sampling and DNA Extraction

A total of 76 mantodean species were sampled in the phylogenetic analysis, representing all spinomantodean (all extant Mantodea excluding Chaeteessidae) families (Table S1). Additionally, four blattodean species—*Mastotermes darwiniensis*, *Coptotermes lacteus*, *Cryptocercus kyebangensis*, and *Blattella germanica*—were used as outgroups. The 23 newly sequenced species in the present study were collected in the field (see Table S2 for detailed information) and preserved in 100% ethanol at −20 °C at the Entomological Museum of China Agricultural University for DNA extraction. Genomic DNA was extracted from the thoracic and coxal muscle tissues of the specimens using the DNeasy Blood and Tissue kit (Qiagen, Hilden, Germany) following the manufacturer’s protocol.

### 4.2. Molecular Marker Generation

We used fifteen mitochondrial genes (13 protein-coding genes (PCGs) and two rRNAs) and three nuclear genes (*H3*, *18S* rRNA, and *28S* rRNA) as molecular markers for phylogenetic analyses (Table S1). These gene fragments were obtained from whole-genome next-generation sequencing (NGS) yielded by this study, and transcriptomic data retrieved from Sequence Read Archive (SRA) or GenBank databases.

Single-species Illumina Truseq libraries were prepared for the newly sequenced species with an average insert size of 400 bp and sequenced using the Illumina HiSeq 2500 platform (Illumina, San Diego, CA, USA) at BerryGenomics (Beijing, China) with 150 bp paired-end reads. After trimming adapters and removing low-quality and short reads, high-quality reads were used for de novo assembly using IDBA-UD [61] with minimum and maximum k-values of 45 and 145 bp, respectively. For sequences derived from transcriptomic data, the raw reads downloaded from SRA datasets were quality-filtered using Fastp v.0.12.4 [62] and assembled using Geneious v.10.1.3 [63] with the default settings. The partial Cytochrome Oxidase I (*COI*) sequence of the related species was used as bait to identify the mitogenomic assemblies while partial *H3*, *18S* rRNA, and *28S* rRNA sequences were used to identify corresponding nuclear gene fragments of the target species from assembled reads in Geneious v.10.1.3 [63]. All of the bait sequences were retrieved from GenBank.

Twenty-three completed mitogenomes and four nearly completed mitogenomes (*Mantoida* sp., *Brunneria borealis*, *Acontista multicolor*, and *Empusa pennata*) were generated from our genomic assemblies and SRA assemblies, respectively, following the above pipelines. These mitogenomes were then annotated using Mito Z [64] and further corrected in Geneious v.10.1.3 [63] by aligning the sequences with those of homologous genes of other Mantodea species. The remaining 53 mitochondrial sequences used in phylogenetic analyses were retrieved from the GenBank database (Table S1).

We identified three nuclear gene fragments of 21 species from our genomic assemblies and obtained additional data from SRA assemblies (three *H3*s, two *18S* rRNAs, and two *28S* rRNAs) or GenBank (45 *H3*s, 50 *18S* rRNAs, and 51 *28S* rRNAs). For species that lacked nuclear genes, we obtained gene sequences of closely related congeneric species from GenBank (Table S1). The total numbers of sampled *H3*, *18S* rRNA, and *28S* rRNA sequences were 69, 73, and 74, respectively. The average coverage of nuclear genes for the species used in the phylogenetic analyses was approximately 90%.

### 4.3. Sequence Alignment, Concatenation, and Analyses

Each PCG was individually aligned with codon-based multiple alignments using the MAFFT algorithm [65] on the TranslatorX online platform with the L-INS-i strategy and default settings [66]. Sequences of rRNA genes were separately aligned using MAFFT v.7.0, an online server that employs the G-INS-i strategy [65]. MEGA v.7.0 [67] was used to check, concatenate, and output the alignments. Four datasets were prepared for phylogenetic analyses: (i) the 13 PCGs and two rRNA genes of the mitogenomes (PCGRNA, 12,687 bp); (ii) the 13 PCGs with third codon positions removed and two rRNA genes of the mitogenomes (PCG12RNA, 9071 bp); (iii) the 13 PCGs and two rRNA genes of the mitogenomes with three nuclear genes (NGPCGRNA, 16,830 bp); and (iv) the 13 PCGs with third codon positions removed and two rRNA genes of mitogenomes with three nuclear genes (NGPCG12RNA, 13,214 bp).

The base composition of the concatenated datasets was analyzed using MEGA v.7.0 [67]. DnaSP v.5.0 [68] was used to estimate the rate of synonymous substitutions (Ks) and the rate of non-synonymous substitutions (Ka) of the 13 PCGs to evaluate the evolutionary rate. *Blattella germanica* was chosen as the reference species to estimate the Ka and Ks values.

AliGROOVE [69] was used with the default sliding window size to visualize the heterogeneity of sequence divergence in PCGRNA, PCG12 (PCG dataset with the third codon positions removed), and PCG3 (PCG dataset with the third codon positions only) datasets. Indels in the nucleotide datasets were treated as ambiguous, and a BLOSUM62 matrix was used as the default amino acid substitution matrix. This metric established pairwise sequence distances between individual terminals or subclades with terminals outside the focal group. The distances were then compared with the distances over the entire data matrix. The reference species was chosen as *Rhombodera zhangi* to represent the overall sequence similarity.

### 4.4. Phylogenetic Analyses

Bayesian inference (BI), maximum likelihood (ML), and maximum parsimony (MP) methods were used in the phylogenetic analyses based on four datasets (PCGRNA, PCG12RNA, NGPCGRNA, and NGPCG12RNA).

We used PhyloBayes MPI v.1.5a [33] for BI analyses under the site-heterogeneous mixture model CAT + GTR with a discrete gamma distribution and four rate categories. Two Markov Monte Carlo chains were run independently until the sampled trees reached satisfactory convergence (maxdiff less than 0.1). After the initial 25% of trees were discarded as burn-in, a consensus tree was computed from the remaining trees combined from two runs.

Three partitioning strategies were used for ML analyses of each dataset (no partitions, gene partitions, and codon partitions for mitochondrial PCGs). The optimal partitioning scheme and the best substitution model for each partition were selected using ModelFinder [70] in IQ-TREE v.1.6.12 [71] (Table S3). Phylogenetic trees were constructed using the IQ-TREE web server using an ultrafast bootstrap approximation approach with 10,000 replicates. The branch lengths of the topology based on the PCGRNA dataset were calculated using IQ-TREE for further analyses.

TNT v.1.5 [72] was applied to conduct MP analyses, with the “New Technology search” method under default parameters and by setting random seed as 0 and the initial driven search level at 15. Bootstrapping analyses were conducted with 1000 bootstrap replicates to calculate nodal support.

### 4.5. Phylogenetic Hypothesis Testing

Tree topology tests were performed using the one-sided Kishino–Hasegawa (KH) test [73], Shimodaira–Hasegawa (SH) test [74], weighted KH (WKH), weighted SH (WSH), and approximately unbiased (AU) tests [75], with 10,000 resamplings using the RELL method [76] under the GTR + G model in IQ-TREE. Four heterogeneous-based hypotheses from PhyloBayes and two prior familial hypotheses by Svenson and Rodrigues [14] and Schwarz and Roy [2] were input into IQ-TREE and tested under various analytic approaches mentioned above.

Because the inferred positions of Chroicopteroidea, Nanomantoidea, Gonypetoidea, and Haanioidea were unstable in both BI and ML trees in our various datasets, we conducted FcLM [6,39] to test the alternative phylogenetic hypotheses of the four superfamilies and determine whether the relationship in our best tree inferred from the strict datasets was influenced by the confounding mitogenome signals. The input datasets including five clusters: (i) Chroicopteroidea; (ii) Nanomantoidea; (iii) Haanioidea; (iv) Gonypetoidea; and (v) the remaining species used in phylogenetic analyses set with the option ‘IGNORED’. Four datasets (PCG12RNA, NGPCG12RNA, PCGRNA, and NGPCGRNA) were individually analyzed under the best-fit model according to the BIC criteria in IQ-TREE (Table S3).

## 5. Conclusions

Our study constructed a family-level mantodean phylogeny using larger datasets with mitogenomes and nuclear genes. We found that multiple tRNA duplications are prevalent across the whole Spinomantodea, with the first detection of *trnL2* duplication in Mantodea. The analysis of sequence features first confirmed the lineage-specific compositional heterogeneity among mantodean mitogenomes, demonstrating that model selection is critical for resolving mantodean phylogeny. The supplementation of the nuclear genes could also improve mitochondrial phylogenomic topologies. The final results selected by topology tests and FcLM supported most cladistic relationships in the current taxonomic system, placing Metallyticoidea nested within Artimantodea, and recovered epaphroditoidean lineages Epaphroditidae as the sister group to the rest of Cernomantodea, whereas Majangidae was a sister group to Mantidae. The new cladistic hypotheses were also proposed for the early branching of Cernomantodea as ((Chroicopteroidea + Haanioidea) + (‘Gonypetoidea’ + Nanomantoidea)), and the derived lineage of Heteromantodea as ((Miomantoidea + Hoplocoryphoidea + Eremiaphiloidea) + Promantidea). We thus suggest that further studies should be conducted using the site-heterogeneous model, filtering optimal strategies, increasing taxa sampling, especially on basal and controversial clades, and using diversified datasets to better resolve Mantodea phylogeny.

## Figures and Tables

**Table 1:** Recovery of key nodes obtained using PhyloBayes (BI), IQ-TREE (ML), and TNT (MP).

		BI				ML												MP			
	Dataset	PR	P12R	NPR	NP12R	PR			P12R			NPR			NP12R			PR	P12R	NPR	NP12R
	Partition					N	G	C	N	G	C	N	G	C	N	G	C				
Schizomantodea		0.98	0.97	0.97	0.93	0.92	NS	0.92	0.95	1	0.92	0.97	NS	0.99	0.99	1.00	0.98	400	333	457	381
Heteromantodea *		0.97	0.96	0.99	0.95	1.00	0.99	1.00	0.98	0.91	1.00	0.99	1.00	1.00	1.00	1.00	1.00	NS	NS	NS	NS
Amerimantodea		NS	NS	0.44	NS	NS	NS	NS	NS	NS	NS	NS	NS	NS	NS	NS	NS	NS	NS	NS	NS
Mantidea *		0.47	NS	0.9	0.48	NS	NS	NS	NS	NS	NS	NS	0.49	NS	NS	NS	NS	NS	NS	NS	NS
Promantidea *		1.00	0.96	1.00	1.00	0.99	0.99	0.99	0.98	0.91	0.96	1.00	1.00	1.00	1.00	1.00	1.00	NS	NS	NS	NS
Haanioidea		NS	0.42	NS	0.6	NS	NS	NS	NS	NS	NS	NS	NS	NS	NS	NS	0.58	NS	NS	NS	NS
Nanomantoidea		1.00	1.00	1.00	1.00	1.00	1.00	1.00	1.00	1.00	1.00	1.00	1.00	1.00	1.00	1.00	1.00	22	22	17	20
Eremiaphiloidea		1.00	1.00	1.00	1.00	1.00	1.00	1.00	1.00	1.00	1.00	1.00	1.00	1.00	1.00	1.00	1.00	37	25	42	24
Chroicopteroidea		1.00	1.00	1.00	1.00	1.00	1.00	1.00	1.00	1.00	1.00	1.00	1.00	1.00	1.00	1.00	1.00	61	32	66	39
Hymenopoidea		1.00	1.00	1.00	1.00	NS	0.93	NS	0.91	0.85	0.84	0.50	0.91	NS	0.94	1.00	0.94	27	NS	28	14
MHEC		0.97	0.96	0.98	0.92	NS	0.50	NS	0.62	0.94	0.89	0.72	0.85	0.57	0.66	0.61	0.90	NS	NS	16	7
MECE		0.85	0.85	0.98	0.99	NS	NS	NS	NS	NS	NS	NS	NS	NS	0.78	NS	NS	34	NS	7	13
GNC		0.97	0.52	0.99	0.95	NS	NS	NS	NS	NS	NS	NS	0.89	NS	NS	NS	NS	NS	NS	NS	NS
GMC *		1.00	NS	1.00	1.00	NS	NS	NS	NS	NS	NS	NS	1.00	NS	NS	NS	NS	NS	NS	NS	NS

Notes: The clades that failed to recover in all analyses are not included in the table. Abbreviations: N, no partition; G, gene partition; C, codon partition; NS, not supported. PR, PCGRNA dataset; P12R, PCG12RNA dataset; NPR, NGPCGRNA dataset; NP12R, NGPCG12RNA dataset. MHEC, (Miomantoidea + Hoplocoryphoidea + Eremiophiloidea) clade. MECE, (Metallyticoidea + Ceromantodea) clade. GNC, (Gonypetoidea + Nanomantoidea) clade. GMC, (Galinthiadoidea + Mantidea) clade. * indicates the clade in which Majangidae is nested.

**Table 2:** Topology tests for four hypotheses derived from the site-heterogeneous CAT + GTR model in this study and two widely accepted hypotheses from previous studies.

Dataset	Tree	logL	deltaL	bp-RELL	*p*-KH	*p*-SH	*p*-WKH	*p*-WSH	c-ELW	*p*-AU
NGPCG12RNA	PCGRNA	−230,935.7142	78.335	0.0007−	0.0084−	0.0318−	0.0084−	0.0222−	0.000728−	0.00427
	PCG12RNA	−230,888.8728	31.494	0.131+	0.211+	0.337+	0.211+	0.391+	0.131+	0.196+
	NGPCGRNA	−230,857.3788	0	0.497+	0.561+	1+	0.561+	0.818+	0.497+	0.634+
	NGPCG12RNA	−230,861.4544	4.0756	0.371+	0.439+	0.7+	0.439+	0.689+	0.371+	0.541+
NGPCGRNA	PCGRNA	−452,278.8796	104.43	0.0031−	0.004−	0.0171−	0.004−	0.0095−	0.00312−	0.00735−
	PCG12RNA	−452,296.1574	121.71	0.0044−	0.0077−	0.0098−	0.0077−	0.0171−	0.0046−	0.00849−
	NGPCGRNA	−452,174.449	0	0.985+	0.993+	1+	0.993+	1+	0.985+	0.996+
	NGPCG12RNA	−452,253.6602	79.211	0.0075−	0.0071−	0.0704+	0.0071−	0.0168−	0.00728−	0.0114−
PCG12RNA	PCGRNA	−204,317.2383	34.324	0.0839+	0.0858+	0.206+	0.0858+	0.19+	0.0845+	0.0893+
	PCG12RNA	−204,282.914	0	0.915+	0.914+	1+	0.914+	0.987+	0.915+	0.928+
	NGPCGRNA	−204,369.6892	86.775	0.0005−	0.0028−	0.0029−	0.0028−	0.007−	0.000449−	0.0027−
	NGPCG12RNA	−204,361.0502	78.136	0.0005−	0.0013−	0.0068−	0.0013−	0.0027−	0.000508−	0.000323−
PCGRNA	PCGRNA	−422,801.9202	0	0.755+	0.79+	1+	0.79+	0.945+	0.756+	0.844+
	PCG12RNA	−422,828.4959	26.576	0.211+	0.21+	0.384+	0.21+	0.386+	0.21+	0.243+
	NGPCGRNA	−422,848.051	46.131	0.0343−	0.0503+	0.162+	0.0503+	0.116+	0.0346−	0.0621+
	NGPCG12RNA	−422,920.0939	118.17	0−	0.0003−	0.0004−	0.0003−	0.0016−	1.21 × 10^−7^−	5.1 × 10^−5^−
PCGRNA	Schwarz and Roy, 2019 ^1^	−417,699.9417	0	0.996+	0.996+	1+	0.996+	0.996+	0.996+	0.996+
	Svenson and Rodrigues, 2017 ^2^	−417,803.5686	103.63	0.0041−	0.0043−	0.0043−	0.0043−	0.0043−	0.0041−	0.00378−
PCG12RNA	Schwarz and Roy, 2019 ^1^	−202,107.3782	0	0.994+	0.994+	1+	0.994+	0.994+	0.994+	0.995+
	Svenson and Rodrigues, 2017 ^2^	−202,190.6792	83.301	0.0063−	0.0061−	0.0061−	0.0061−	0.0061−	0.00617−	0.00514−
NGPCGRNA	Schwarz and Roy, 2019 ^1^	−447,001.7467	0	0.893+	0.895+	1+	0.895+	0.895+	0.893+	0.889+
	Svenson and Rodrigues, 2017 ^2^	−447,056.1321	54.385	0.107+	0.105+	0.105+	0.105+	0.105+	0.107+	0.111+
NGPCG12RNA	Schwarz and Roy, 2019 ^1^	−228,551.765	0	0.825+	0.825+	1+	0.825+	0.825+	0.825+	0.827+
	Svenson and Rodrigues, 2017 ^2^	−228,585.8363	34.071	0.175+	0.175+	0.175+	0.175+	0.175+	0.175+	0.173+

^1^ Schwarz and Roy, 2019: Recent phylogenetic framework of Mantodea based on multiple pieces of evidence with an emphasis on male genitalia (Majangidae excluded). ^2^ Svenson and Rodrigues, 2017: Nine gene-based (five mitochondrial gene fragments and four nuclear gene fragments) ML phylogenetic tree of Mantodea with all 16 superfamilies included for the first time (Majangidae was excluded). deltaL, LogL difference from the maximal logl in the set. bp-RELL, bootstrap proportion using RELL method. *p*-KH, *p*-value of one-sided KH test. *p*-SH, *p*-value of SH test. *p*-WKH, *p*-value of weighted KH test. *p*-WSH, *p*-value of weighted SH test. c-ELW, expected likelihood weight. *P*-AU, *p*-value of AU test. + denotes a value within the 95% confidence interval. − denotes significant exclusion.

## Data Availability

The fully aligned datasets are accessible through figshare “https://doi.org/10.6084/m9.figshare.21687692 (accessed on 8 December 2022)”. Other data supporting the findings of this study are available within the article and its supplementary materials. GenBank accession numbers are given in Table S1.

## Supplementary Materials

The following supporting information can be downloaded at: https://www.mdpi.com/article/10.3390/ijms241310570/s1.
