# Supplementary material for: Exploring the Mitogenomes of Mantodea: New Insights from Structural Diversity and Higher-Level Phylogenomic Analyses
Source: Int J Mol Sci. 2023 Jun 24;24(13):10570. doi: 10.3390/ijms241310570 (PMC10342016; doi:10.3390/ijms241310570)
Supplement: Supplementary file 1 [file ijms-24-10570-s001.zip › ijms-2411567-supplementary.pdf]

Table S1. Taxa used in the present study and their accession numbers.

**Superfamily**	**Family**	**Species**	**Mitogenome**	***H3***	***18S* rRNA**	***28S* rRNA**
Mantoidoidea	Mantoididae	_Mantoida_ sp.	BK062891	NA	OP962224	OP962222
Metallyticoidea	Metallyticidae	_Metallyticus splendidus_	KY689122	FJ806762	FJ806399	FJ806598
Thespoidea	Thespidae	*_Thesprotiella_ sp.	OP831271	OP831576	OP856578	OP881396
	Angelidae	*_Angela_ sp.	OP831256	OP831556	OP856558	OP881376
	Coptopterygidae	_Brunneria borealis_	BK062890	SRR1811961	KU557277	KU557282
		*_Coptopteryx_ sp.	OP831272	OP831561	OP856563	OP881381
		*_Metilia_ sp.	OP831277	OP831572	OP856574	OP881392
Acanthopoidea	Acanthopidae	_Acontista multicolor_	BK062892	SRR1811954	KU320383 (_A. gracilis_)	KU320487 (_A. gracilis_)
		*_Stenophylla lobivertex_	OP831269	EF384167	EF383589	EF383755
	Liturgusidae	*_Hagiomantis_ sp.	OP831266	OP831568	OP856570	OP881388
	Photinaidae	_Macromantis hyalina_	MT370515	KU507864	KU320416	KU320520
Chroicopteroidea	Chroicopteridae	*_Carvilia saussurii_	OP831259	OP831559	OP856561	OP881379
		*_Gonypetella_ sp.	OP831265	OP831567	OP856569	OP881387
Nanomantoidea	Leptomantellidae	*_Leptomantella xizangensis_	OP831275	NA	NA	NA
		Leptomantella albella	KJ463364	FJ806736	FJ806486	FJ806572
	Amorphoscelidae	_Amorphoscelis singporana_	MT370512	FJ806730	FJ806366	FJ806565
	Nanomantidae	_Eomantis yunnanensis_	KY689138	EF384094 (_E. guttatipennis_)	EF383515 (_E. guttatipennis_)	EF383676 (_E. guttatipennis_)
		Tropidomantis tenera	NC037205	FJ806738	FJ806375	FJ806574
		Pliacanthopus bimaculatus	NC051490	NA	FJ806502 (_P. mantispoides_)	FJ806704 (_P. mantispoides_)
		Sceptuchus simplex	NC037206	EF384113	EF383534	EF383697
		Spilomantis occipitalis	KX091854	FJ806843 (_S. nitens_)	FJ806483 (_S. nitens_)	FJ806685 (_S. nitens_)
Gonypetoidea	Gonypetidae	_Amantis nawai_	NC037203	FJ806722 (_A. reticulata_)	FJ806337 (_A. reticulata_)	FJ806535 (_A. reticulata_)
		Theopompa sp.	KU201313	NA	NA	NA
		Humbertiella nada	NC030264	EF384090 (_H. similis_)	FJ806501 (_H. ocularis_)	FJ806570 (_H. ocularis_)
Epaphroditoidea	Majangidae	*_Brancsikia freyi_	OP831276	OP831558	OP856560	OP881378
	Epaphroditidae	*_Epaphrodita musarum_	OP831278	OP831563	OP856565	OP881383
Haanioidea	Haaniidae	_Caliris_ sp.	KY689126	NA	NA	NA
		Arria pallida	NC051892	FJ806785 (_A._	FJ806422 (_A._	FJ806624 (_A._

				meghalayensis)	*meghalayensis*)	*meghalayensis*)
		Haania sp.	KY689130	FJ806842 (*H. lobiceps*)	FJ806482 (*H. lobiceps*)	FJ806684 (*H. lobiceps*)
Hoplocoryphoidea	Hoplocoryphidae	**Hoplocorypha* sp.	OP831267	OP831569	OP856571	OP881389
Miomantoidea	Miomantidae	**Cilnia humeralis*	OP831261	OP831560	OP856562	OP881380
Galinthiadoidea	Galinthiadidae	**Galinthias amoena*	OP831264	OP831566	OP856568	OP881386
	Eremiaphilidae	*Schizocephala bicornis*	NC037207	EF384087	EF383508	EF383669
		**Eremiaphila* sp.	OP831274	OP831564	OP856566	OP881384
	Amelidae	*Yersinia mexicana*	MW357303	EF384197	EF383620	EF383787
Eremiaphiloidea	Rivetinidae	**Deiphobe yunnanensis*	OP831262	OP831562	OP856564	OP881382
		**Bolivaria brachyptera*	OP831258	OP831557	OP856559	OP881377
	Toxoderidae	*Stenotoxodera porioni*	KY689118	EF384105	EF383526	EF383689
		Paratoxodera polyacantha	NC037697	NA	NA	NA
	Empusidae	**Idolomantis diabolica*	OP831268	OP831570	OP856572	OP881390
		Empusa pennata	BK062893	FJ806752 (*Empusa* sp.)	FJ806389 (*Empusa* sp.)	FJ806588 (*Empusa* sp.)
Hymenopoidea	Hymenopodidae	*Phyllocrania paradoxa*	NC056869	DQ874006	KR360536	KR360574
		Sibylla pretiosa	KY689116	EF384142	FJ806391	FJ806590
		Odontomantis sp.	KY689121	FJ806782 (*O. pulchra*)	FJ806419 (*O. pulchra*)	FJ806621 (*O. pulchra*)
		Anaxarcha zhengi	NC030268	FJ806729 (*A. limbata*)	FJ806365 (*A. limbata*)	FJ806564 (*A. limbata*)
		Hymenopus coronatus	MZ573776	AY491334	EF383476	EF383638
		Theopropus elegans	KY689125	FJ806735	FJ806372	FJ806571
		Creobroter elongata	KX091851	FJ806734 (*Creobroter* sp.)	KR360561	KR360594
		Parablepharis kuhlii	KY689117	NA	NA	NA
		Phyllothelys breve	MT024239	EF384096 (*P. westwoodi*)	EF383517 (*P. westwoodi*)	EF383678 (*P. westwoodi*)
		Ceratomantis saussurii	KX091850	EF384156 (*Ceratomantis* sp.)	EF383578 (*Ceratomantis* sp.)	EF383744 (*Ceratomantis* sp.)
		Hestiasula sp.	KX091855	FJ806825 (*H.*	FJ806464 (*H.*	FJ806665 (*H.*

				masoni)	*masoni*)	*masoni*)
		**Ephestiasula* sp.	OP831273	EF384085 (*Ephestiasula* sp.)	EF383506 (*Ephestiasula* sp.)	EF383667 (*Ephestiasula* sp.)
		**Astyliasula phyllopus*	OP831257	NA	FJ806507	FJ806710
		Psychomantis borneensis	NC045876	FJ806847	KR360558	FJ806689
	Dactylopterygidae	**Theopompella* sp.	OP831270	OP831575	OP856577	OP881395
		Popa spurca	NC056860	KR478678	EF383547	EF383710
		**Euchomenella heteroptera*	OP831263	OP831565	OP856567	OP881385
	Deroplatyidae	*Deroplatys desiccata*	KY689113	FJ806871	FJ806515	FJ806719
		Deroplatys lobata	MT370513	EF384141	EF383563	EF383729
		Deroplatys truncata	MT370514	FJ806872	FJ806516	FJ806720
		Orthodera novaezealandiae	NC056862	EF384078	EF383473	EF383635
		Asiadodis yunnanensis	MN037794	NA	NA	NA
		**Choeradodis rhombicollis*	OP831260	AY491340	EF383481	EF383643
		Mantis religiosa	MN356097	AY491327	EF383471	EF383633
		Statilia maculata	KX900484	FJ806860	FJ806503	FJ806706
Mantoidea		*Tenodera sinensis*	KY689132	GU064792	DQ874190	AY125282
		Sphodromantis lineola	KY689123	GU064762	EF383480	EF383642
		Mekongomantis quinquespinosa	MN267041	OP831571	OP856573	OP881391
	Mantidae	*Tamolanica tamolana*	NC007702	GU064764	EF383483	EF383645
		Hierodula membranacea	NC048984	GU066921	NA	EU414720
		Rhombodera latipronotum	KX091864	OP831573	OP856575	OP881393
		Rhombodera zhangi	MW357299	OP831574	OP856576	OP881394
		Hierodulella sp.	KY689136	FJ806865 (*H. reticulata*)	FJ806509 (*H. reticulata*)	FJ806712 (*H. reticulata*)
		Stagmatoptera biocellata	MW357302	SRR1811993	OP962223	OP962221
		Pseudovates peruviana	MW357301	FJ806839 (*P. denticulata*)	FJ806479 (*P. denticulata*)	FJ806681 (*P. denticulata*)
Outgroups		*Mastotermes darwiniensis*	NC018120	NA	DQ882638	AY125281

	Coptotermes lacteus	KU925211	NA	FJ806332	EU253670
	Cryptocercus kyebangensis	NC030191	KF855884	FJ806324	KP986242
	Blattella germanica	NC012901	MF286993	FJ806322	EU253813

* Taxa newly sequenced in this study. NA, not available.

Table S2. Collecting information for the newly sequenced Mantodea species.

Family	Species	Locality	Collection date
Thespidae	*Thesprotiella* sp.	Lima, Peru	2018-X-3
Angelidae	*Angela* sp.	Lima, Peru	2018-X-3
Coptopterygidae	*Coptopteryx* sp.	Argentina	2019-V-6
Acanthopidae	*Metilia* sp.	Lima, Peru	2018-X-3
	Stenophylla lobivertex	Lima, Peru	2018-X-3
Liturgusidae	*Hagiomantis* sp.	Lima, Peru	2021-XII-16
Chroicopteridae	*Carvilia saussurii*	Etosha, Halali, Namibia	2018-III-8
	Gonypetella sp.	Kibale, Uganda	2018-II-19
Leptomantellidae	*Leptomantella xizangensis*	Motuo, Xizang, China	2014-VII-25
Majangidae	*Brancsikia freyi*	Madagascar	2021-VII-7
Epaphroditidae	*Epaphrodita musarum*	Caribbean	2019-IV-4
Hoplocoryphidae	*Hoplocorypha* sp.	Kamanjab, Namibia	2018-III-9
Miomantidae	*Cilnia humeralis*	South Africa	2017-X-19
Galinthiadidae	*Galinthias amoena*	South Africa	2017-X-19
Eremiaphilidae	*Eremiaphila* sp.	Western Asia	2018-V-21
Rivetinidae	*Deiphobe yunnanensis*	Chuxiong, Yunnan, China	2019-VIII-20
	Bolivaria brachyptera	Yili, Xinjiang, China	2018-IX-3
Empusidae	*Idolomantis diabolica*	Tanzania	2021-XI-6
Hymenopodidae	*Ephestiasula* sp.	Sabah, Malaysia	2015-X-20
	Astyliasula phyllopus	Sabah, Malaysia	2016-X-15
Dactylopterygidae	*Theopompella* sp.	Gabon, Ndjole	2019-XI-29
Deroplatyidae	*Euchomenella heteroptera*	Sabah, Malaysia	2016-X-14
Mantidae	*Choeradodis rhombicollis*	Lima, Peru	2021-XII-12

Table S3. Optimal partitioning scheme and substitution model for datasets used in ML analysis.

Dataset and Partition type	Subset position	Best Model
PCGRNA-no partition		GTR+F+I+G4
PCGRNA-gene partition	P1:(ATP6)	GTR+F+I+G4
	P2:(ATP8)	HKY+F+G4
	P3:(COI)	GTR+F+I+G4
	P4:(COII)	GTR+F+I+G4
	P5:(COIII)	GTR+F+I+G4
	P6:(CYTB)	GTR+F+I+G4
	P7:(ND1)	TIM+F+I+G4
	P8:(ND2)	TIM+F+I+G4
	P9:(ND3)	GTR+F+I+G4
	P10:(ND4)	TIM+F+I+G4
	P11:(ND4L)	GTR+F+I+G4
	P12:(ND5)	TIM+F+I+G4
	P13:(ND6)	GTR+F+I+G4
	P14:(12S rRNA)	GTR+F+I+G4
	P15:(16S rRNA)	GTR+F+I+G4
PCGRNA-codon partition	P1:(ATP6_pos1)	GTR+F+I+G4
	P2:(ATP6_pos2)	TPM3u+F+I+G4
	P3:(ATP6_pos3)	TN+F+I+G4
	P4:(ATP8_pos1)	TIM2+F+I+G4
	P5:(ATP8_pos2)	TVM+F+I+G4
	P6:(ATP8_pos3)	K3Pu+F+G4
	P7:(COI_pos1)	GTR+F+I+G4
	P8:(COI_pos2)	TVM+F+I+G4
	P9:(COI_pos3)	TIM2+F+I+G4
	P10:(COII_pos1)	GTR+F+I+G4
	P11:(COII_pos2)	GTR+F+I+G4

	P12:(COII_pos3)	TPM2+F+I+G4
	P13:(COIII_pos1)	GTR+F+I+G4
	P14:(COIII_pos2)	TIM+F+I+G4
	P15:(COIII_pos3)	TPM2u+F+ASC+G4
	P16:(CYTB_pos1)	GTR+F+I+G4
	P17:(CYTB_pos2)	GTR+F+I+G4
	P18:(CYTB_pos3)	TIM2+F+I+G4
	P19:(ND1_pos1)	TPM2+F+I+G4
	P20:(ND1_pos2)	GTR+F+I+G4
	P21:(ND1_pos3)	TPM3+F+G4
	P22:(ND2_pos1)	GTR+F+I+G4
	P23:(ND2_pos2)	TVM+F+I+G4
	P24:(ND2_pos3)	TVM+F+G4
	P25:(ND3_pos1)	TIM2+F+I+G4
	P26:(ND3_pos2)	TVM+F+I+G4
	P27:(ND3_pos3)	TPM2u+F+ASC+G4
	P28:(ND4_pos1)	TVM+F+I+G4
	P29:(ND4_pos2)	TVM+F+I+G4
	P30:(ND4_pos3)	K3Pu+F+I+G4
	P31:(ND4L_pos1)	TPM2u+F+G4
	P32:(ND4L_pos2)	TIM+F+G4
	P33:(ND4L_pos3)	K3Pu+F+G4
	P34:(ND5_pos1)	TIM2+F+I+G4
	P35:(ND5_pos2)	GTR+F+I+G4
	P36:(ND5_pos3)	TPM2u+F+G4
	P37:(ND6_pos1)	TIM2+F+G4
	P38:(ND6_pos2)	GTR+F+I+G4
	P39:(ND6_pos3)	TPM2+F+ASC+G4
	P40:(12S rRNA)	GTR+F+I+G4

	P41:(16S rRNA)	GTR+F+I+G4
PCG12RNA-no partition		GTR+F+I+G4
PCG12RNA-gene partition	P1:(ATP6)	GTR+F+I+G4
	P2:(ATP8)	HKY+F+I+G4
	P3:(COI)	GTR+F+I+G4
	P4:(COII)	GTR+F+I+G4
	P5:(COIII)	GTR+F+I+G4
	P6:(CYTB)	GTR+F+I+G4
	P7:(ND1)	TIM+F+I+G4
	P8:(ND2)	TIM+F+I+G4
	P9:(ND3)	TIM+F+I+G4
	P10:(ND4)	GTR+F+I+G4
	P11:(ND4L)	GTR+F+G4
	P12:(ND5)	TIM+F+I+G4
	P13:(ND6)	TIM+F+I+G4
	P14:(12S rRNA)	GTR+F+I+G4
	P15:(16S rRNA)	GTR+F+I+G4
PCG12RNA-codon partition	P1:(ATP6_pos1)	GTR+F+I+G4
	P2:(ATP6_pos2)	TIM3+F+I+G4
	P3:(ATP8_pos1)	TIM3+F+I+G4
	P4:(ATP8_pos2)	TPM2+F+I+G4
	P5:(COI_pos1)	GTR+F+I+G4
	P6:(COI_pos2)	TVM+F+I+G4
	P7:(COII_pos1)	GTR+F+I+G4
	P8:(COII_pos2)	GTR+F+I+G4
	P9:(COIII_pos1)	GTR+F+I+G4
	P10:(COIII_pos2)	TIM+F+I+G4
	P11:(CYTB_pos1)	GTR+F+I+G4
	P12:(CYTB_pos2)	GTR+F+I+G4

	P13:(ND1_pos1)	TPM2+F+I+G4
	P14:(ND1_pos2)	GTR+F+I+G4
	P15:(ND2_pos1)	GTR+F+I+G4
	P16:(ND2_pos2)	TVM+F+I+G4
	P17:(ND3_pos1)	TIM2+F+I+G4
	P18:(ND3_pos2)	TVM+F+I+G4
	P19:(ND4_pos1)	TVM+F+I+G4
	P20:(ND4_pos2)	GTR+F+I+G4
	P21:(ND4L_pos1)	TIM2+F+G4
	P22:(ND4L_pos2)	TIM+F+G4
	P23:(ND5_pos1)	TIM2+F+I+G4
	P24:(ND5_pos2)	GTR+F+I+G4
	P25:(ND6_pos1)	TIM2+F+G4
	P26:(ND6_pos2)	GTR+F+I+G4
	P27:(12S rRNA)	GTR+F+I+G4
	P28:(16S rRNA)	GTR+F+I+G4
NGPCGRNA-no partition		GTR+F+I+G4
NGPCGRNA-gene partition	P1:(ATP6)	GTR+F+I+G4
	P2:(ATP8)	HKY+F+G4
	P3:(COI)	GTR+F+I+G4
	P4:(COII)	GTR+F+I+G4
	P5:(COIII)	GTR+F+I+G4
	P6:(CYTB)	GTR+F+I+G4
	P7:(ND1)	TIM+F+I+G4
	P8:(ND2)	TIM+F+I+G4
	P9:(ND3)	GTR+F+I+G4
	P10:(ND4)	TIM+F+I+G4
	P11:(ND4L)	GTR+F+I+G4
	P12:(ND5)	TIM+F+I+G4

	P13:(ND6)	GTR+F+I+G4
	P14:(12S rRNA)	GTR+F+I+G4
	P15:(16S rRNA)	GTR+F+I+G4
	P16:(H3)	TIM+F+I+G4
	P17:(18S rRNA)	TNe+I+G4
	P18:(28S rRNA)	GTR+F+I+G4
NGPCGRNA-codon partition	P1:(ATP6_pos1)	GTR+F+I+G4
	P2:(ATP6_pos2)	TPM3u+F+I+G4
	P3:(ATP6_pos3)	TN+F+I+G4
	P4:(ATP8_pos1)	TN+F+I+G4
	P5:(ATP8_pos2)	TVM+F+I+G4
	P6:(ATP8_pos3)	HKY+F+G4
	P7:(COI_pos1)	GTR+F+I+G4
	P8:(COI_pos2)	TVM+F+I+G4
	P9:(COI_pos3)	TIM2+F+I+G4
	P10:(COII_pos1)	GTR+F+I+G4
	P11:(COII_pos2)	GTR+F+I+G4
	P12:(COII_pos3)	TPM2u+F+I+G4
	P13:(COIII_pos1)	GTR+F+I+G4
	P14:(COIII_pos2)	TIM+F+I+G4
	P15:(COIII_pos3)	TPM2u+F+ASC+G4
	P16:(CYTB_pos1)	GTR+F+I+G4
	P17:(CYTB_pos2)	GTR+F+I+G4
	P18:(CYTB_pos3)	TIM2+F+I+G4
	P19:(ND1_pos1)	TPM2u+F+I+G4
	P20:(ND1_pos2)	GTR+F+I+G4
	P21:(ND1_pos3)	TPM3+F+G4
	P22:(ND2_pos1)	GTR+F+I+G4
	P23:(ND2_pos2)	TVM+F+I+G4

	P24:(ND2_pos3)	TVM+F+G4
	P25:(ND3_pos1)	TIM2+F+I+G4
	P26:(ND3_pos2)	TVM+F+I+G4
	P27:(ND3_pos3)	TVM+F+ASC+G4
	P28:(ND4_pos1)	TVM+F+I+G4
	P29:(ND4_pos2)	TVM+F+I+G4
	P30:(ND4_pos3)	K3Pu+F+I+G4
	P31:(ND4L_pos1)	TPM2u+F+G4
	P32:(ND4L_pos2)	TIM+F+G4
	P33:(ND4L_pos3)	TPM3u+F+G4
	P34:(ND5_pos1)	TIM2+F+I+G4
	P35:(ND5_pos2)	GTR+F+I+G4
	P36:(ND5_pos3)	TPM2u+F+G4
	P37:(ND6_pos1)	TIM2+F+I+G4
	P38:(ND6_pos2)	GTR+F+I+G4
	P39:(ND6_pos3)	TPM2+F+ASC+G4
	P40:(12S rRNA)	GTR+F+I+G4
	P41:(16S rRNA)	GTR+F+I+G4
	P42:(H3)	TIM+F+I+G4
	P43:(18S rRNA)	TNe+I+G4
	P44:(28S rRNA)	GTR+F+I+G4
NGPCG12RNA-no partition		TVM+F+I+G4
NGPCG12RNA-gene partition	P1:(ATP6)	GTR+F+I+G4
	P2:(ATP8)	HKY+F+I+G4
	P3:(COI)	GTR+F+I+G4
	P4:(COII)	TIM2+F+I+G4
	P5:(COIII)	GTR+F+I+G4
	P6:(CYTB)	GTR+F+I+G4
	P7:(ND1)	TIM+F+I+G4

	P8:(ND2)	TIM+F+I+G4
	P9:(ND3)	TIM+F+I+G4
	P10:(ND4)	GTR+F+I+G4
	P11:(ND4L)	GTR+F+G4
	P12:(ND5)	TIM+F+I+G4
	P13:(ND6)	TIM+F+I+G4
	P14:(12S rRNA)	GTR+F+I+G4
	P15:(16S rRNA)	GTR+F+I+G4
	P16:(H3)	TIM+F+I+G4
	P17:(18S rRNA)	TNe+I+G4
	P18:(28S rRNA)	GTR+F+I+G4
NGPCG12RNA-codon partition	P1:(ATP6_pos1)	GTR+F+I+G4
	P2:(ATP6_pos2)	TPM3+F+I+G4
	P3:(ATP8_pos1)	TN+F+I+G4
	P4:(ATP8_pos2)	TPM2u+F+I+G4
	P5:(COI_pos1)	GTR+F+I+G4
	P6:(COI_pos2)	TVM+F+I+G4
	P7:(COII_pos1)	GTR+F+I+G4
	P8:(COII_pos2)	GTR+F+I+G4
	P9:(COIII_pos1)	GTR+F+I+G4
	P10:(COIII_pos2)	TIM+F+I+G4
	P11:(CYTB_pos1)	GTR+F+I+G4
	P12:(CYTB_pos2)	GTR+F+I+G4
	P13:(ND1_pos1)	TPM2u+F+I+G4
	P14:(ND1_pos2)	GTR+F+I+G4
	P15:(ND2_pos1)	GTR+F+I+G4
	P16:(ND2_pos2)	TVM+F+I+G4
	P17:(ND3_pos1)	TIM2+F+I+G4
	P18:(ND3_pos2)	TVM+F+I+G4

	P19:(ND4_pos1)	TVM+F+I+G4
	P20:(ND4_pos2)	GTR+F+I+G4
	P21:(ND4L_pos1)	TPM2u+F+G4
	P22:(ND4L_pos2)	TIM+F+G4
	P23:(ND5_pos1)	TIM2+F+I+G4
	P24:(ND5_pos2)	GTR+F+I+G4
	P25:(ND6_pos1)	TIM2+F+G4
	P26:(ND6_pos2)	GTR+F+I+G4
	P27:(12S rRNA)	GTR+F+I+G4
	P28:(16S rRNA)	GTR+F+I+G4
	P29:(H3)	TN+F+I+G4
	P30:(18S rRNA)	TNe+I+G4
	P31:(28S rRNA)	GTR+F+I+G4

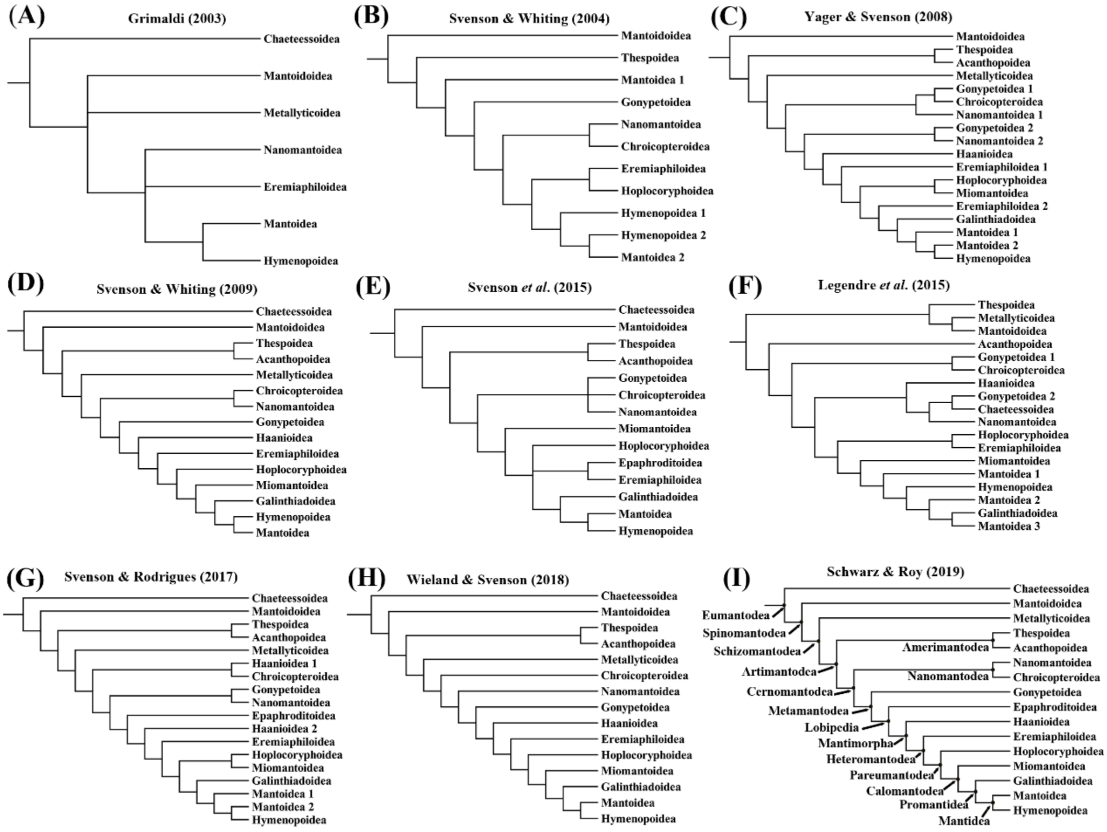

Figures S1. Phylogenetic hypotheses of Mantodea published in the 21st century. (A) Phylogeny based on the morphology of fossil and extant species; (B) Maximum likelihood (ML) and maximum parsimony (MP) analyses based on five genes; (C) ML analysis based on seven genes (Chaeteessoidea was excluded from this topology owing to the data contamination problem indicated by the authors, Svenson and Whiting, 2009); (D) ML analysis inferred from nine genes; (E) Total evidence Bayesian inference (BI) phylogeny with an emphasis on Hymenopoidea; (F) ML analysis based on six genes; (G) ML analysis based on nine genes; (H) Cladistic summary of mantodean phylogeny (Epaphroditoidea unresolved); (I) The most recent taxonomic framework of Mantodea. Superfamily annotation followed the system of Schwarz and Roy (2019).

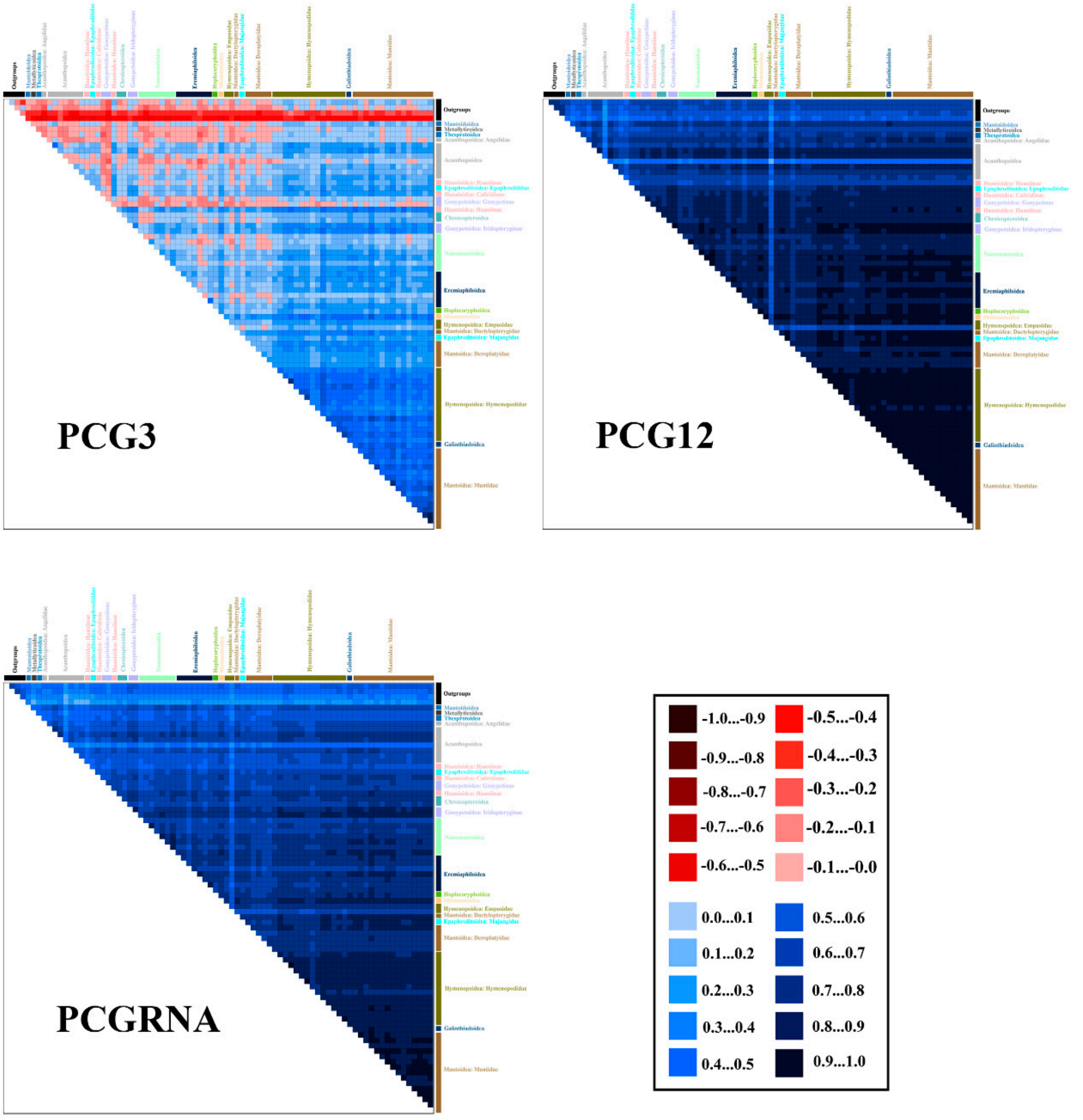

Figures S2. Heterogeneous sequence divergence within mantodean mitogenomic datasets. The mean similarity score between sequences is represented by a colored square, which can vary between -1 if distances are very different from the average for the entire data matrix (red coloring) to +1 for distances that match the average for the entire matrix (blue coloring).

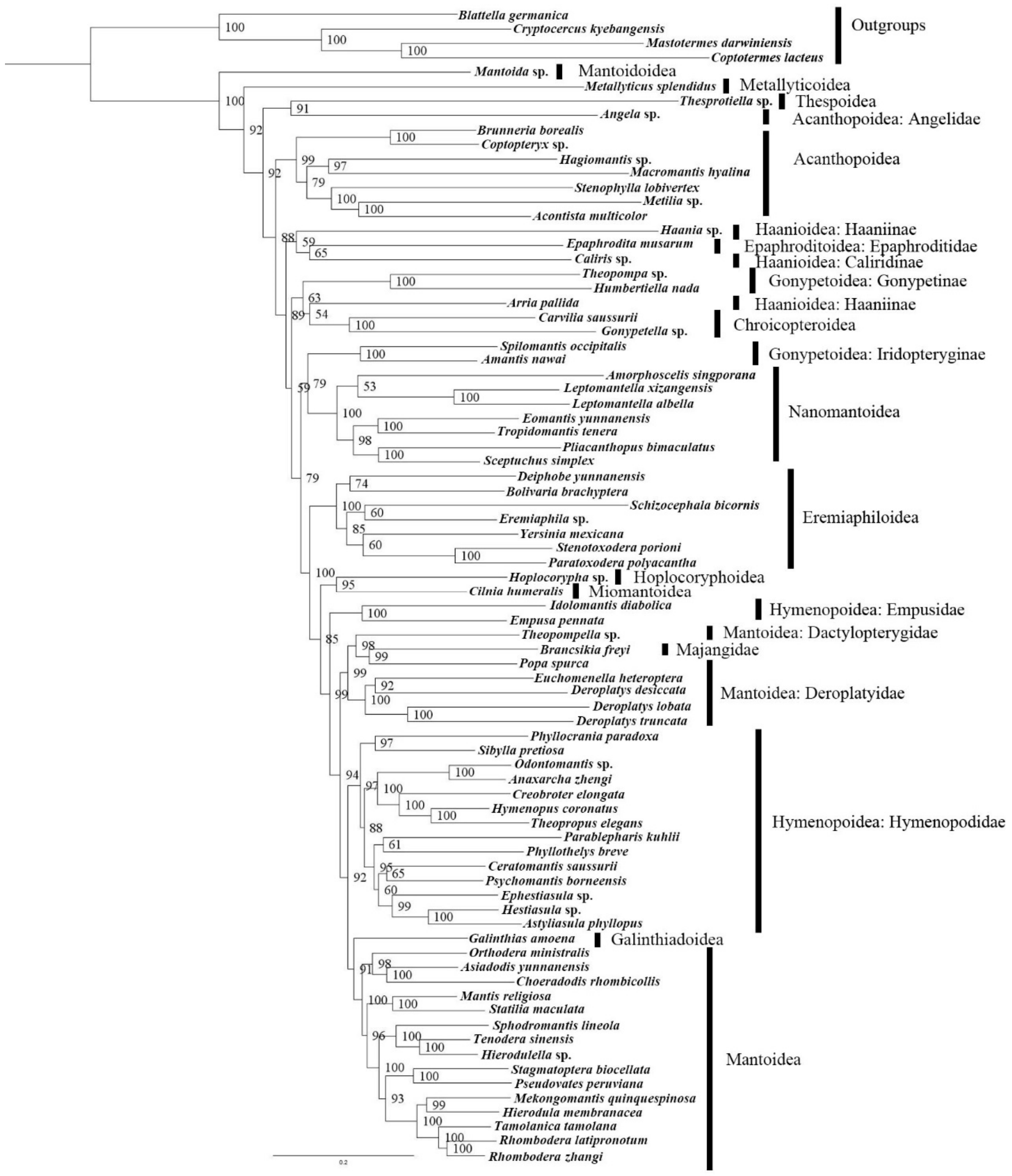

Figures S3. Phylogenetic tree inferred from ML analysis of PCGRNA without data partition under the site-homogeneous model. Bootstrap values of ML analyses were labeled at each node.

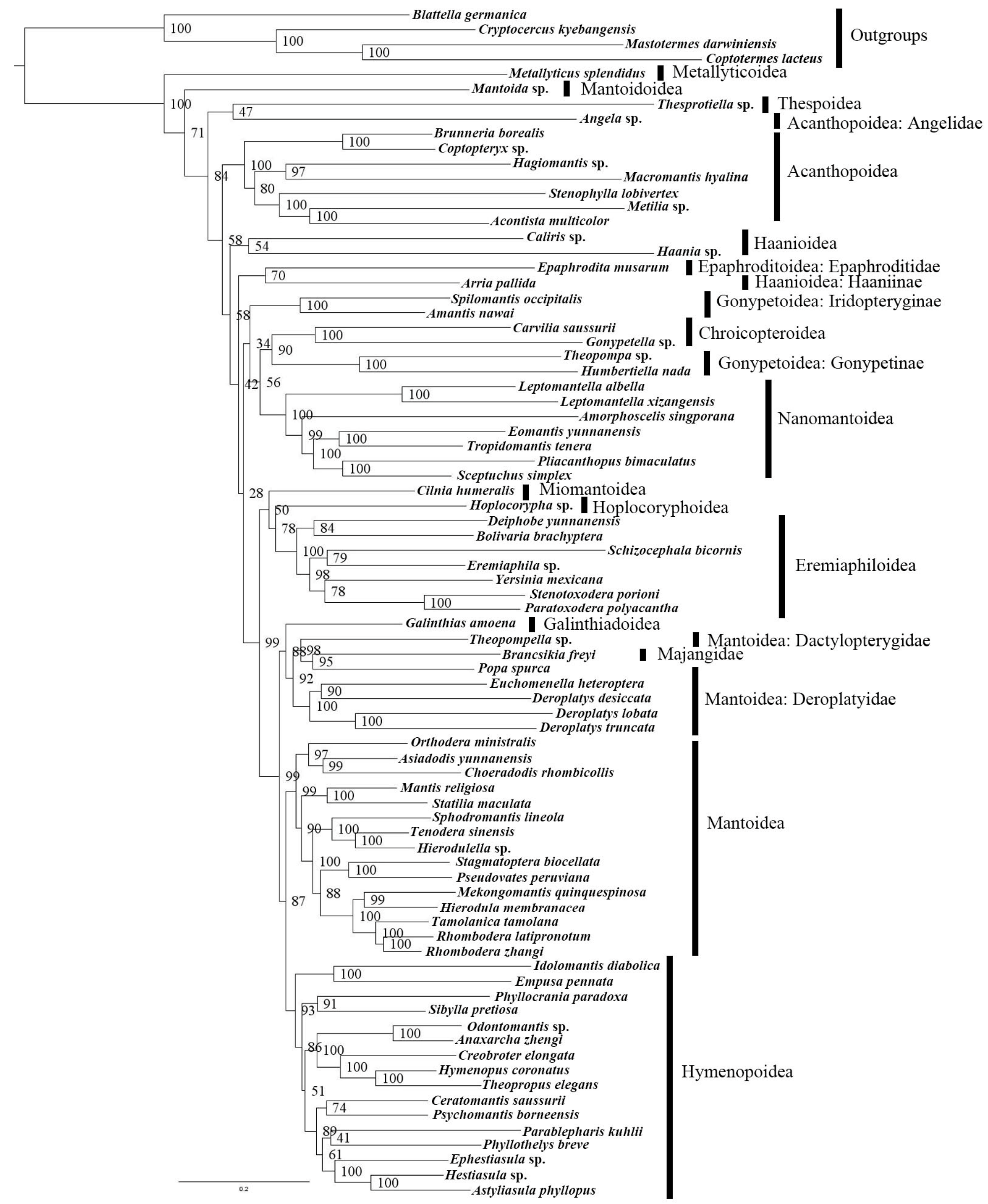

Figures S4. Phylogenetic tree inferred from ML analysis of PCGRNA with gene partition under the site-homogeneous model. Bootstrap values of ML analyses were labeled at each node.

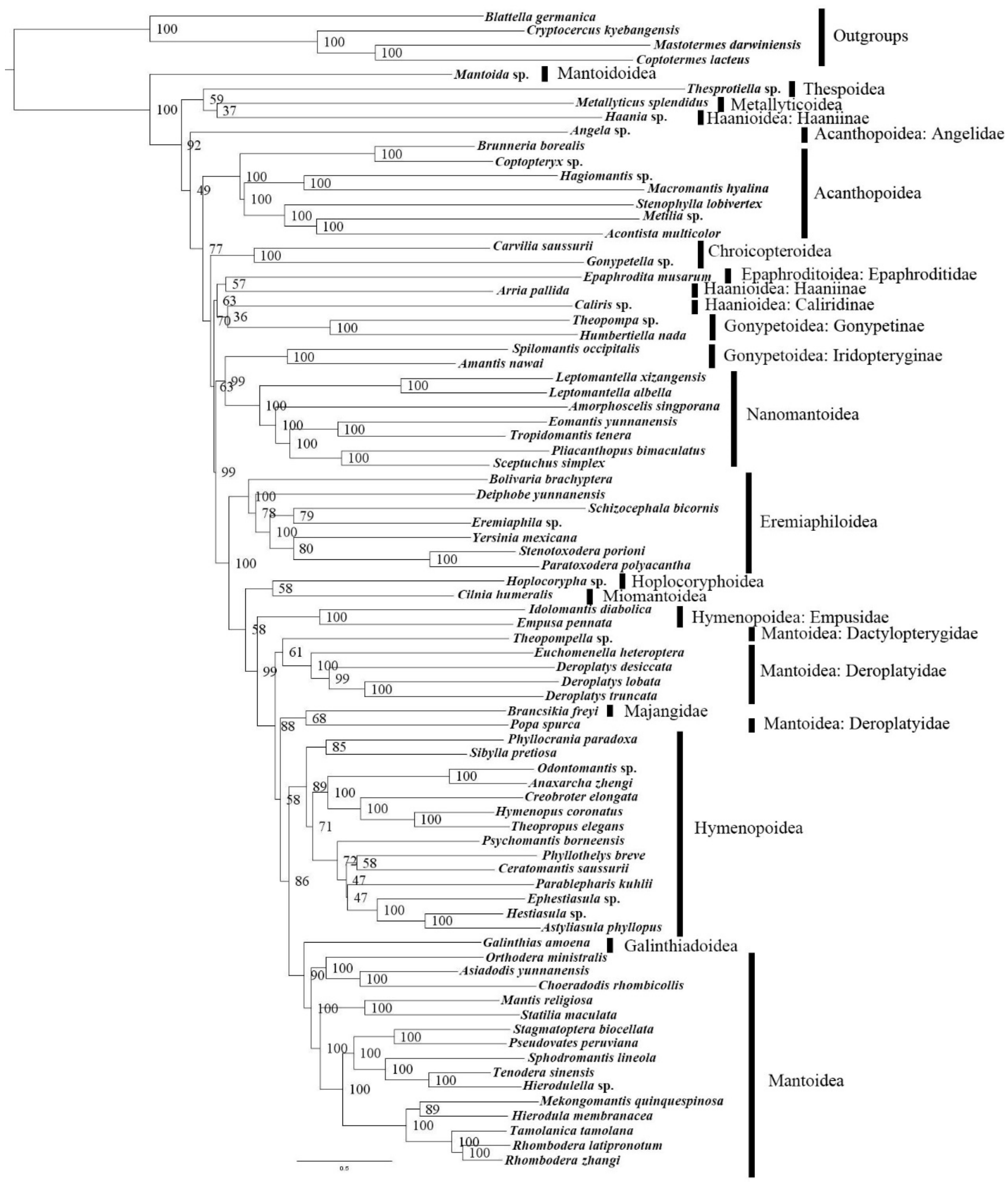

Figures S5. Phylogenetic tree inferred from ML analysis of PCGRNA with codon partition under the site-homogeneous model. Bootstrap values of ML analyses were labeled at each node.

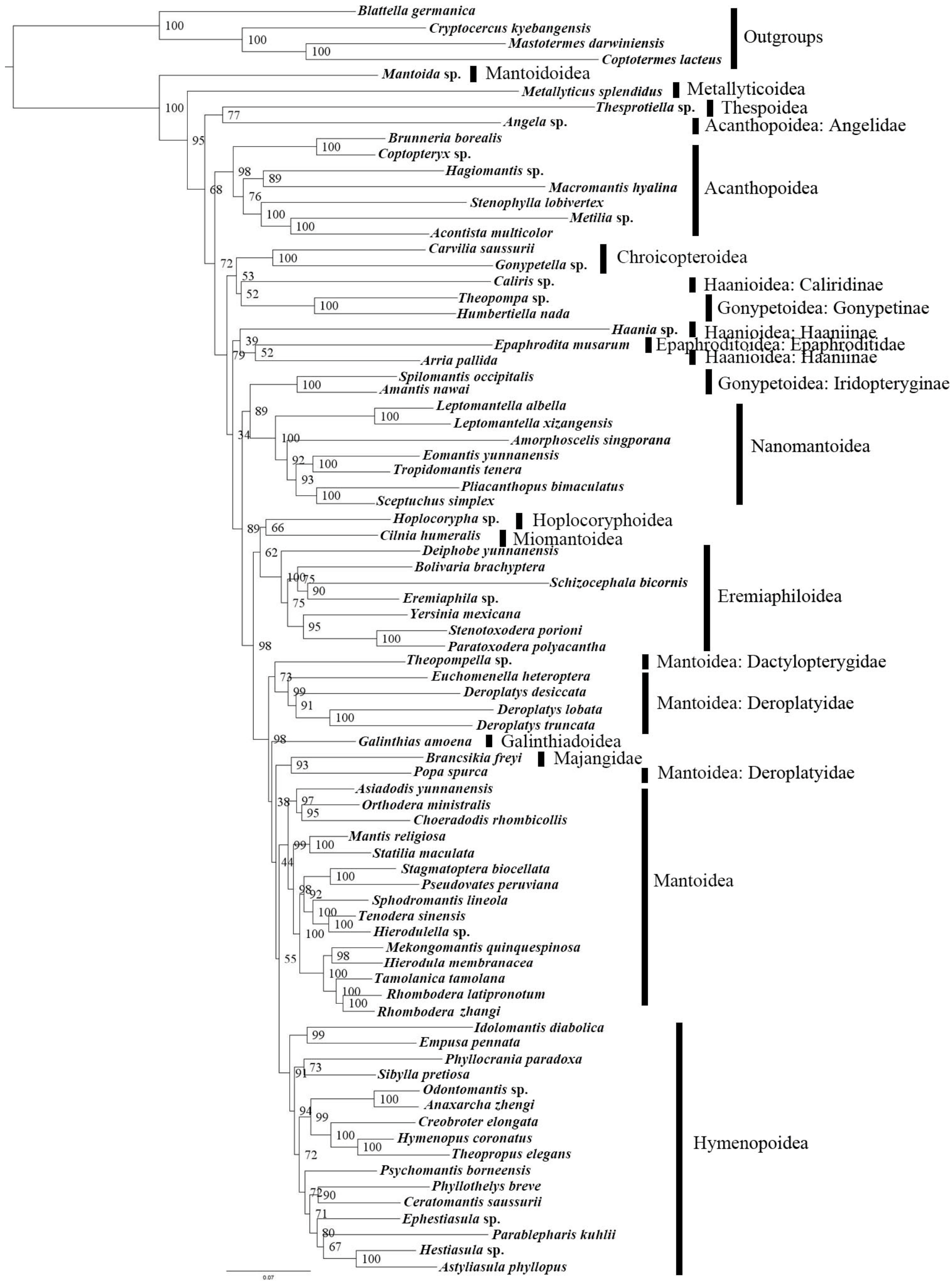

Figures S6. Phylogenetic tree inferred from ML analysis of PCG12RNA without data partition under the site-homogeneous model. Bootstrap values of ML analyses were labeled at each node.

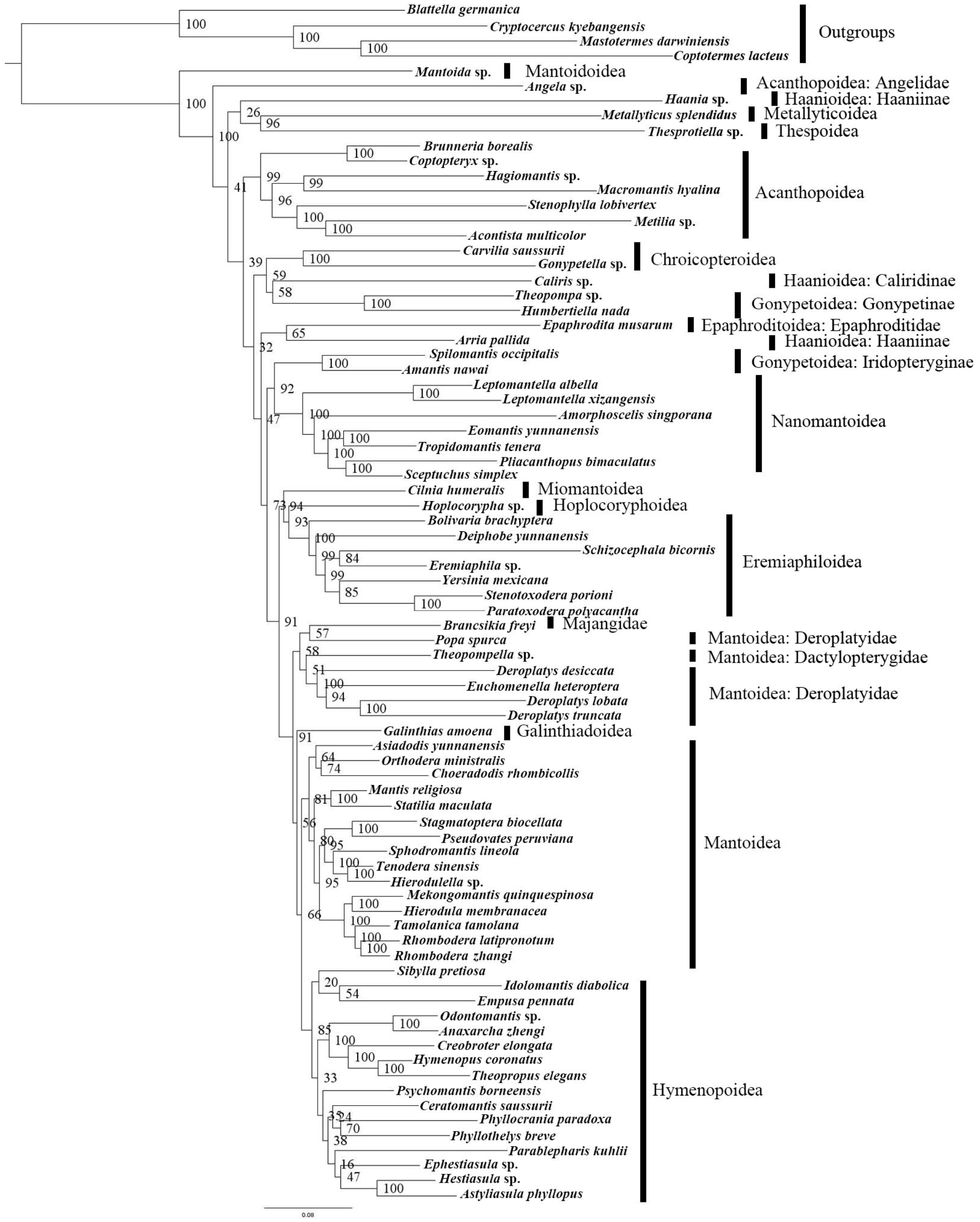

Figures S7. Phylogenetic tree inferred from ML analysis of PCG12RNA with gene partition under the site-homogeneous model. Bootstrap values of ML analyses were labeled at each node.

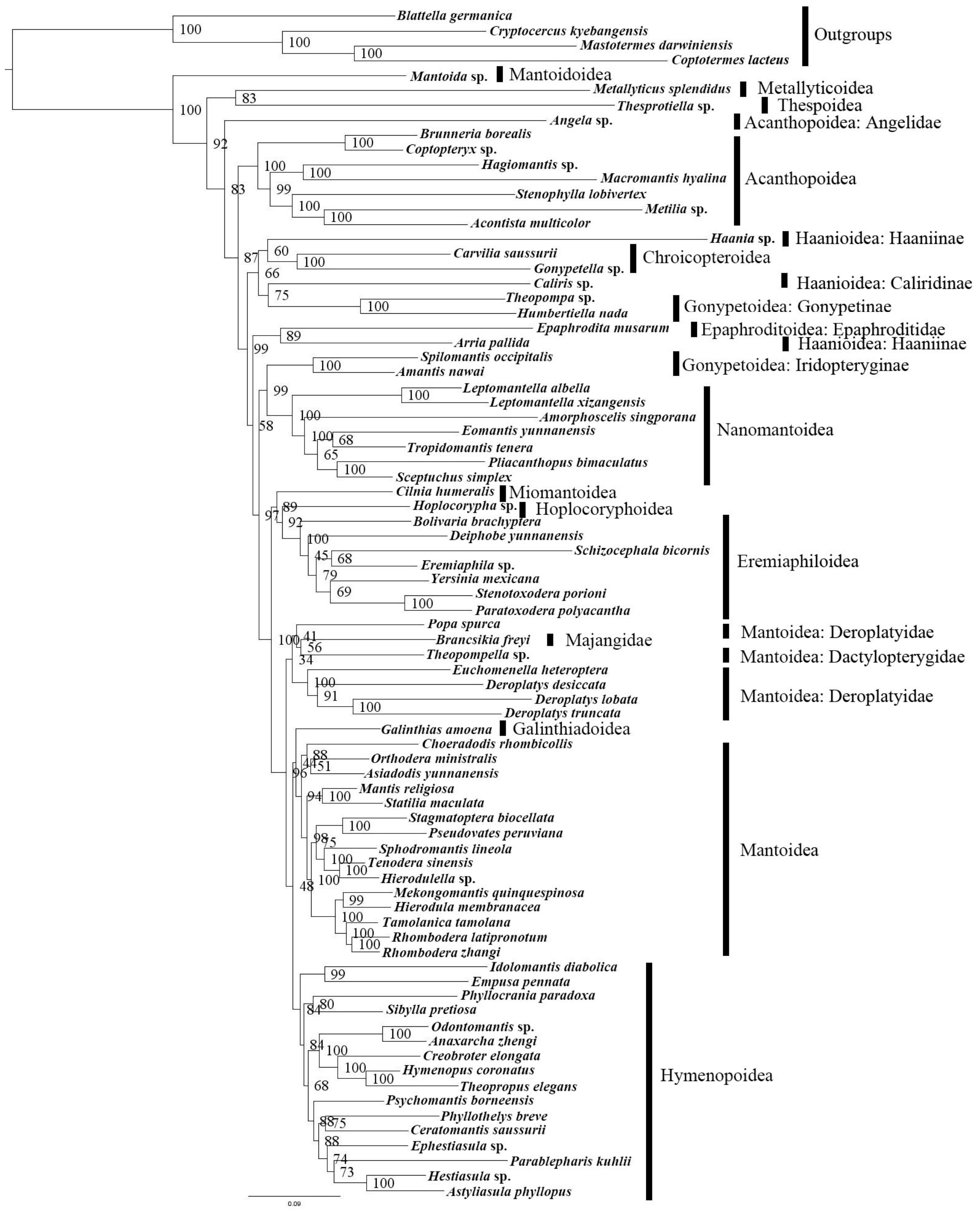

Figures S8. Phylogenetic tree inferred from ML analysis of PCG12RNA with codon partition under the site-homogeneous model. Bootstrap values of ML analyses were labeled at each node.

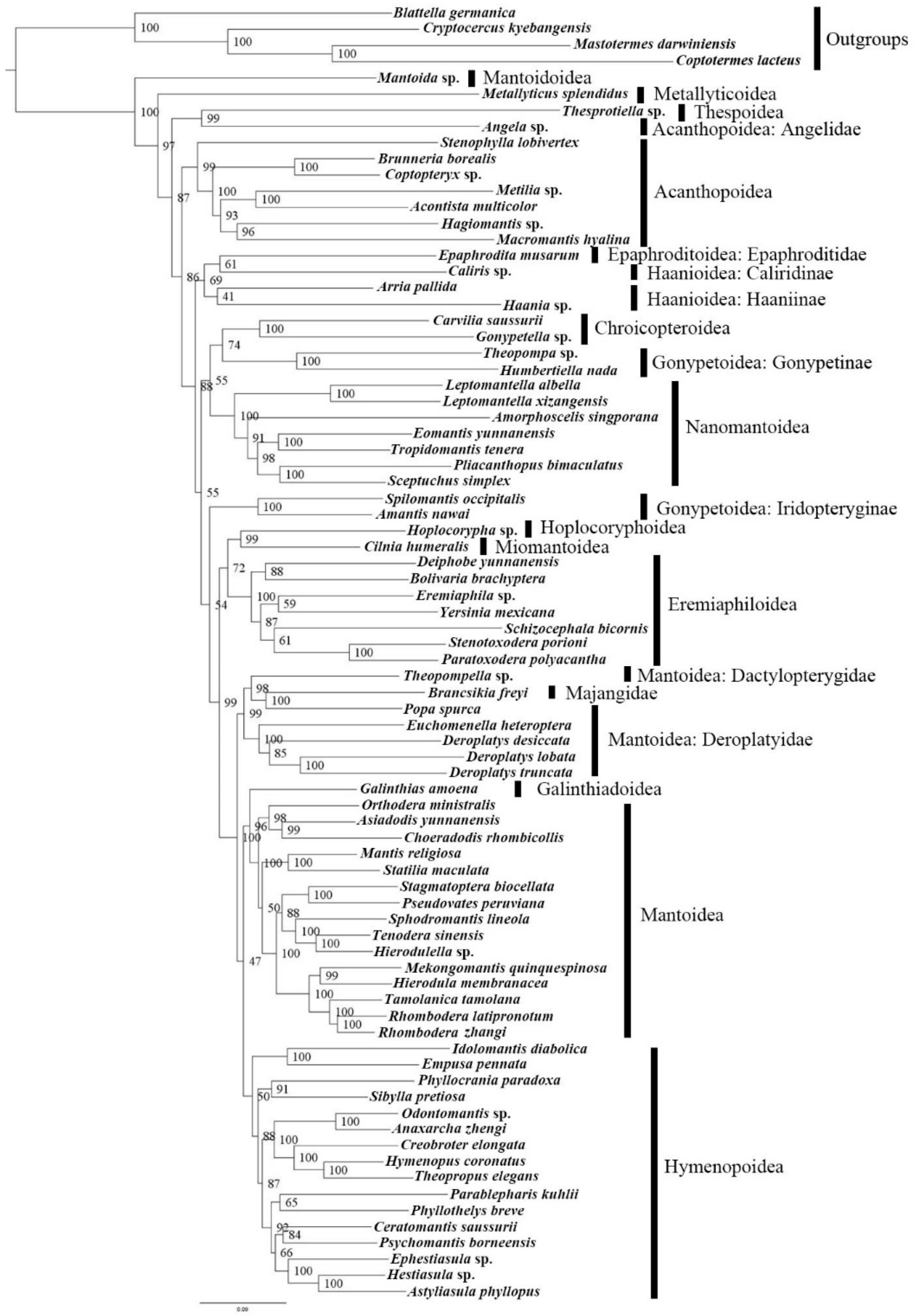

Figures S9. Phylogenetic tree inferred from ML analysis of NGPCGRNA without data partition under the site-homogeneous model. Bootstrap values of ML analyses were labeled at each node.

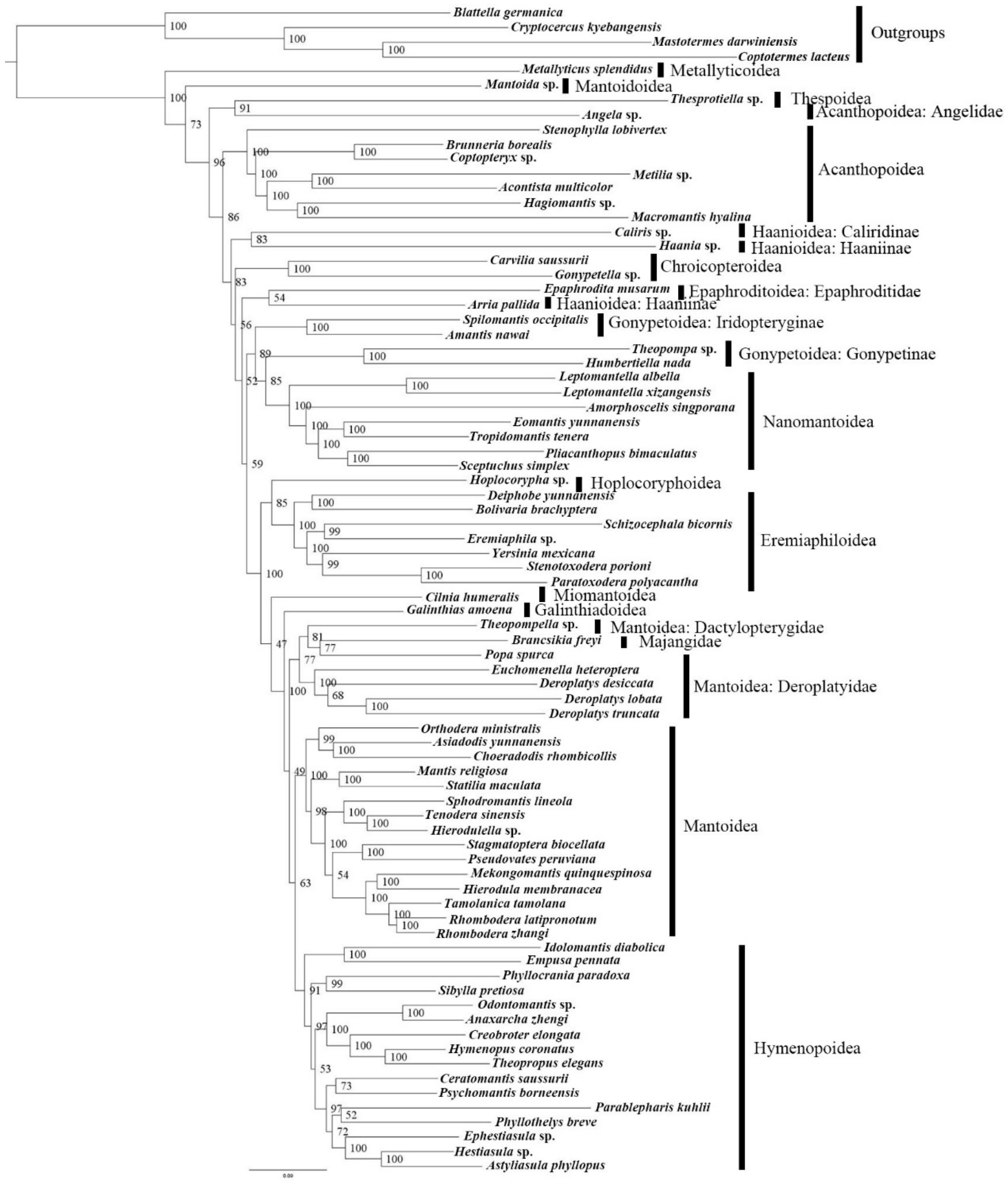

Figures S10. Phylogenetic tree inferred from ML analysis of NGPCGRNA with gene partition under the site-homogeneous model.. Bootstrap values of ML analyses were labeled at each node.

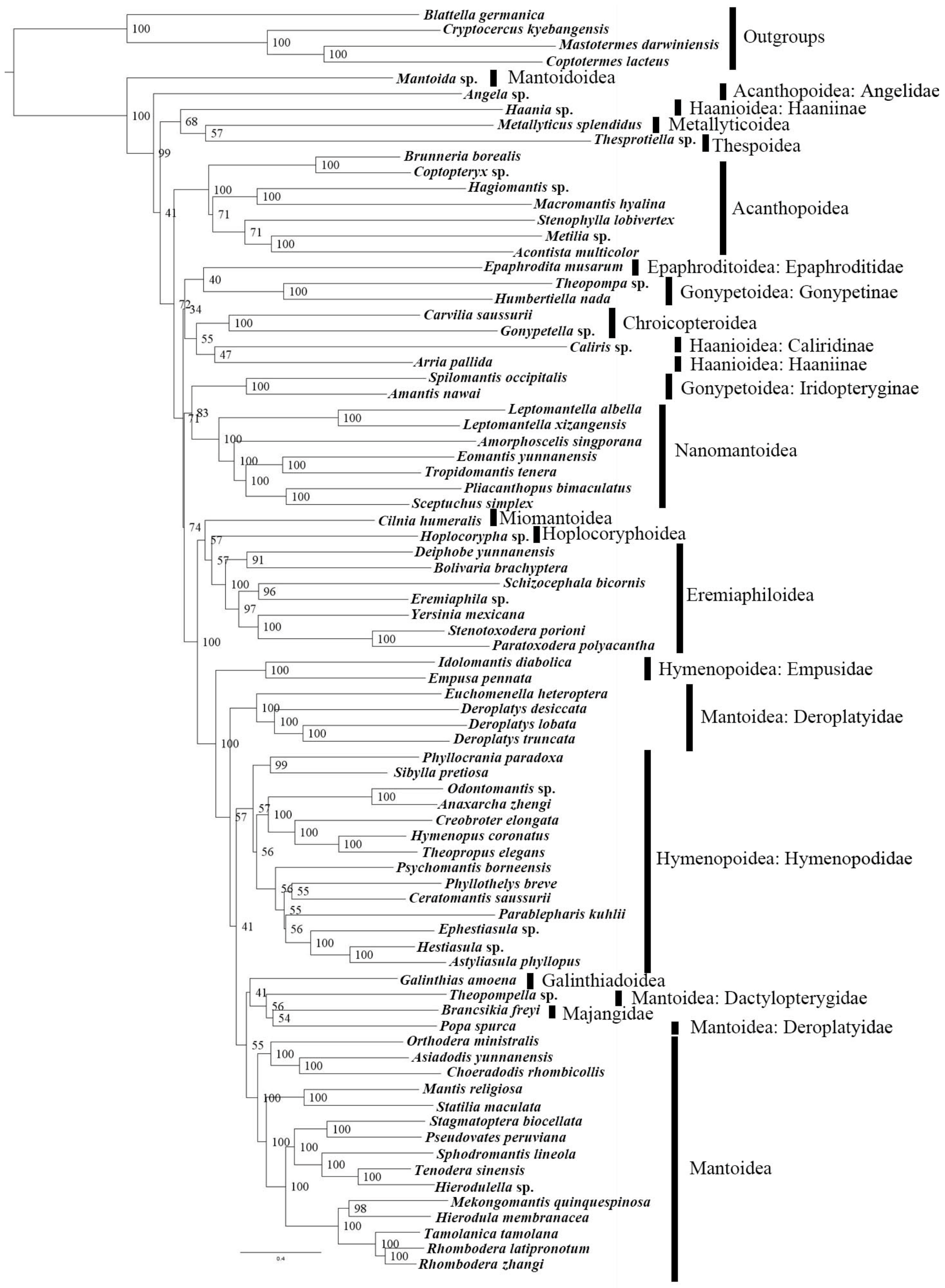

Figures S11. Phylogenetic tree inferred from ML analysis of NGPCGRNA with codon partition under the site-homogeneous model. Bootstrap values of ML analyses were labeled at each node.

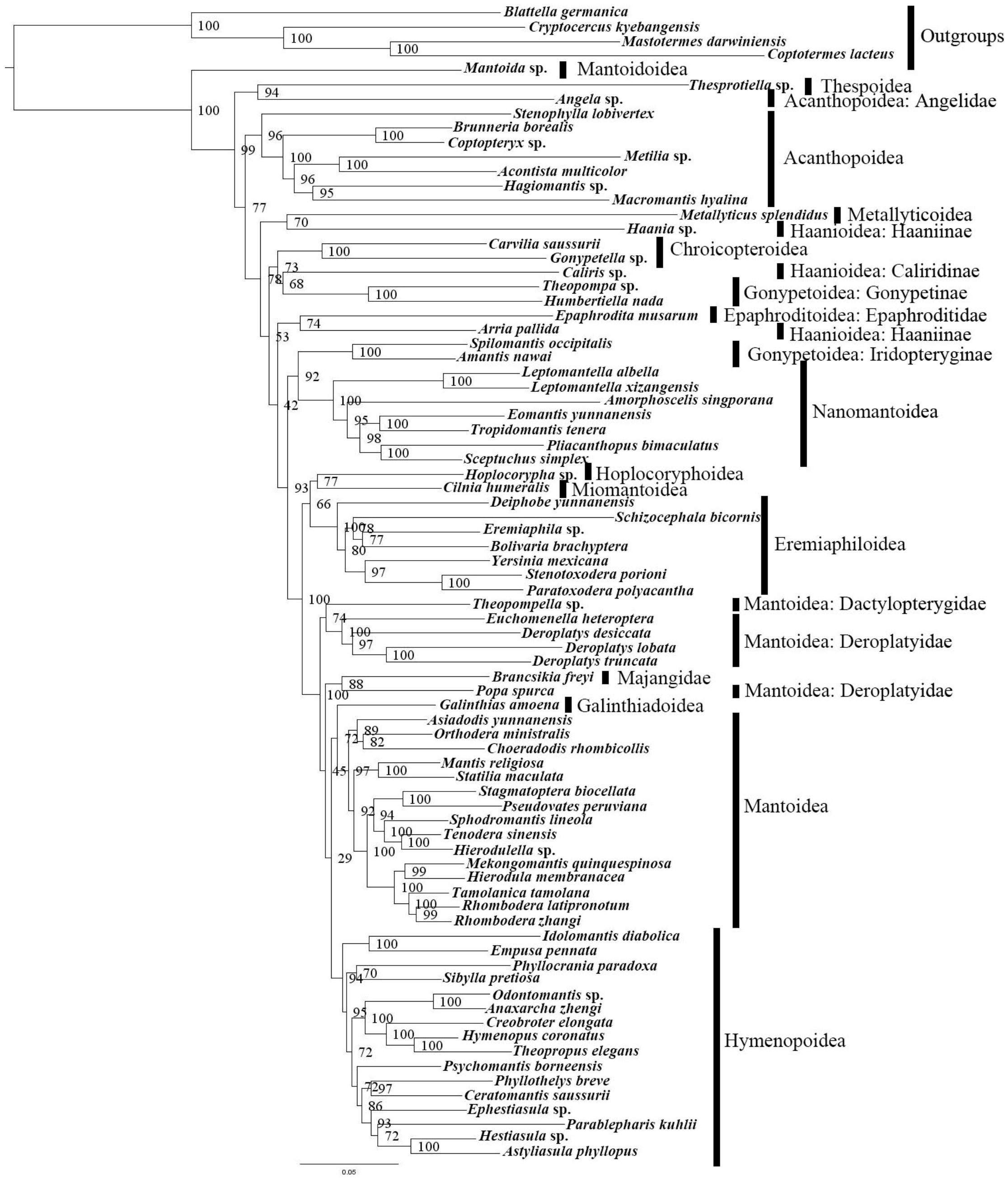

Figures S12. Phylogenetic tree inferred from ML analysis of NGPCG12RNA without data partition under the site-homogeneous model. Bootstrap values of ML analyses were labeled at each node.

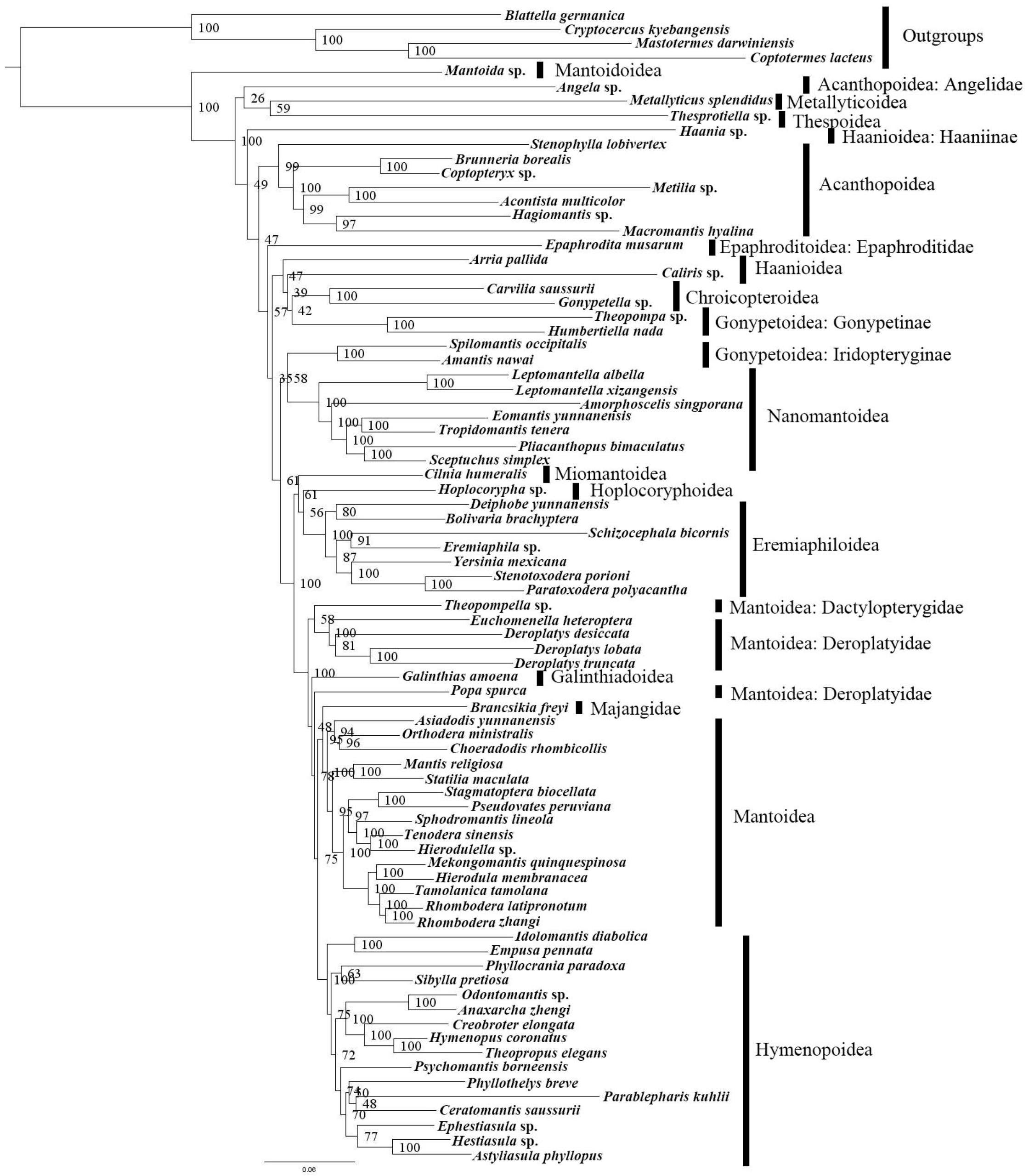

Figures S13. Phylogenetic tree inferred from ML analysis of NGPCG12RNA with gene partition under the site-homogeneous model. Bootstrap values of ML analyses were labeled at each node.

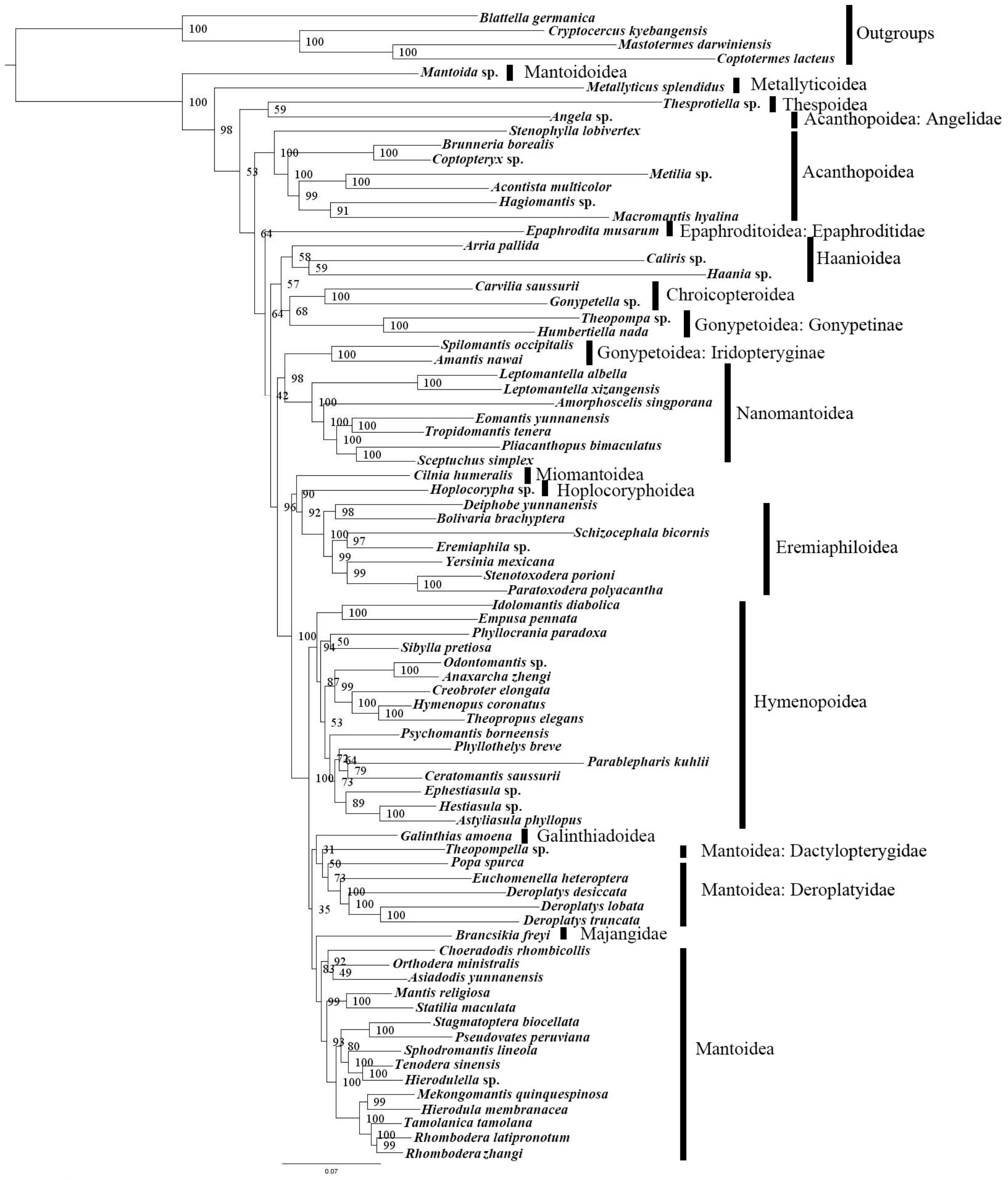

Figures S14. Phylogenetic tree inferred from the ML analysis of NGPCG12RNA with codon partition under the site-homogeneous model. Bootstrap values of ML analyses were labeled at each node.

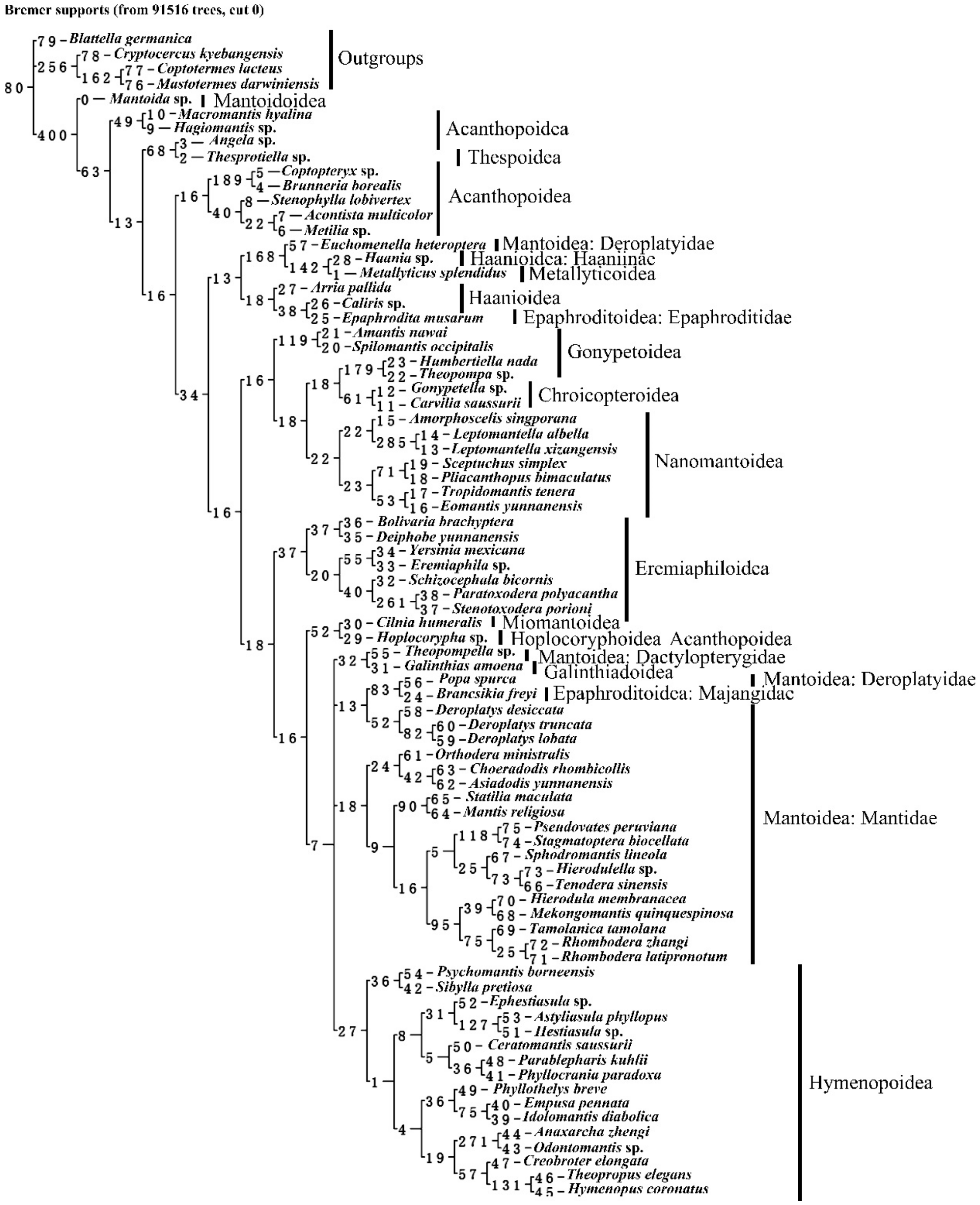

Figures S15. Phylogenetic tree inferred from MP analysis of PCGRNA. Bremer support values of MP analyses were labeled at each node.

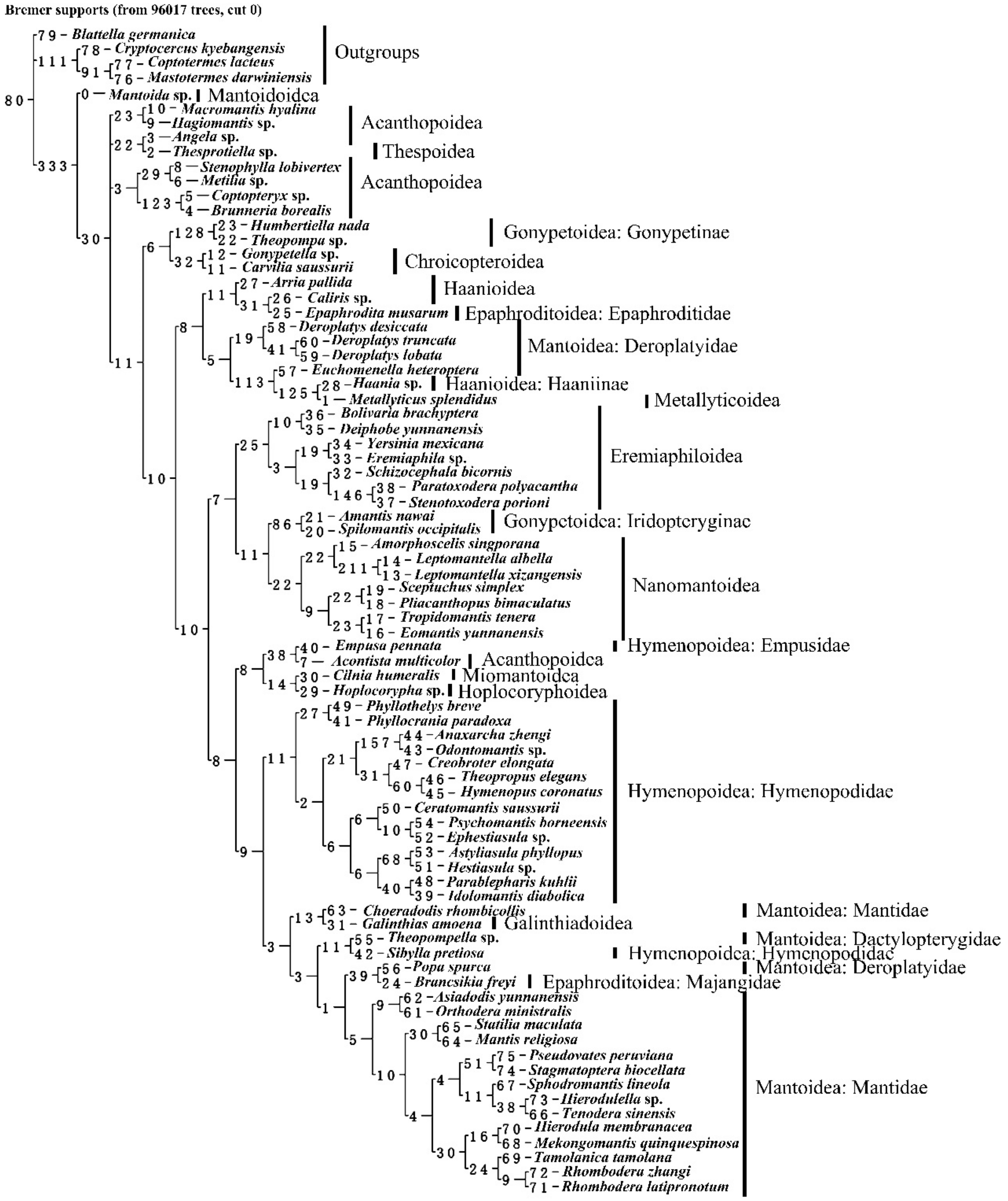

Figures S16. Phylogenetic tree inferred from MP analysis of PCG12RNA. Bremer support values of MP analyses were labeled at each node.

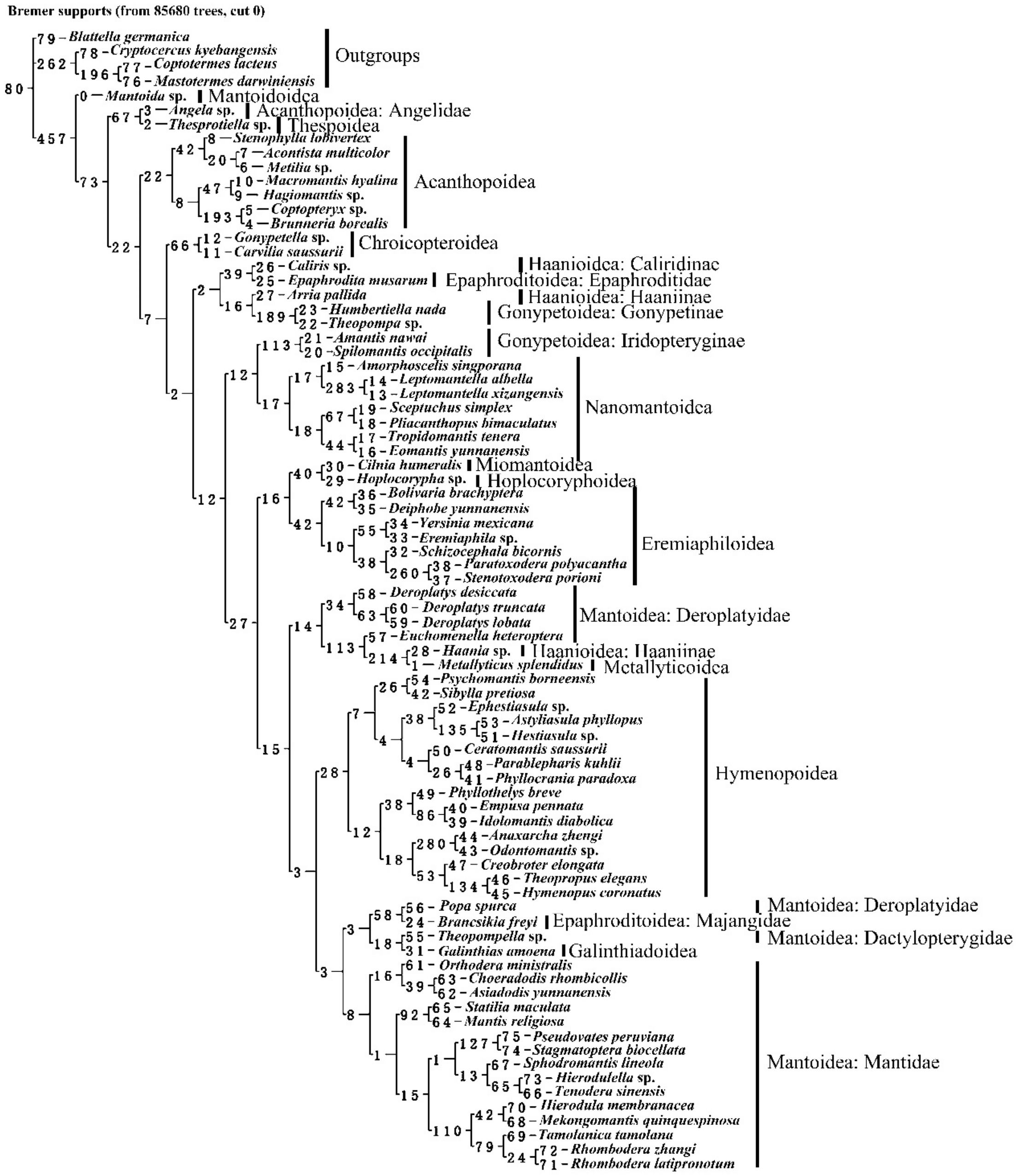

Figures S17. Phylogenetic tree inferred from MP analysis of NGPCGRNA. Bremer support values of MP analyses were labeled at each node.

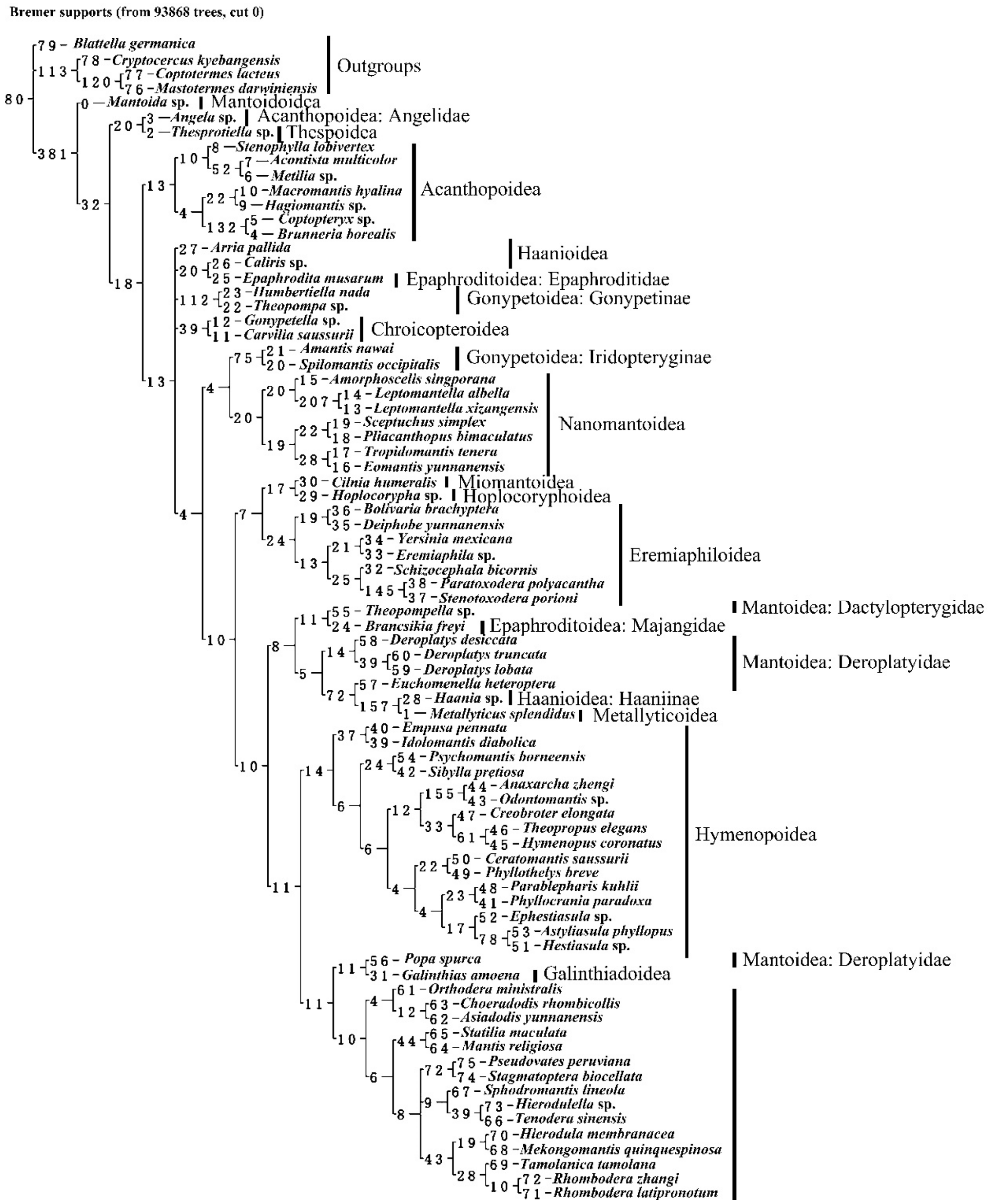

Figures S18. Phylogenetic tree inferred from MP analysis of NGPCG12RNA. Bremer support values of MP analyses were labeled at each node.

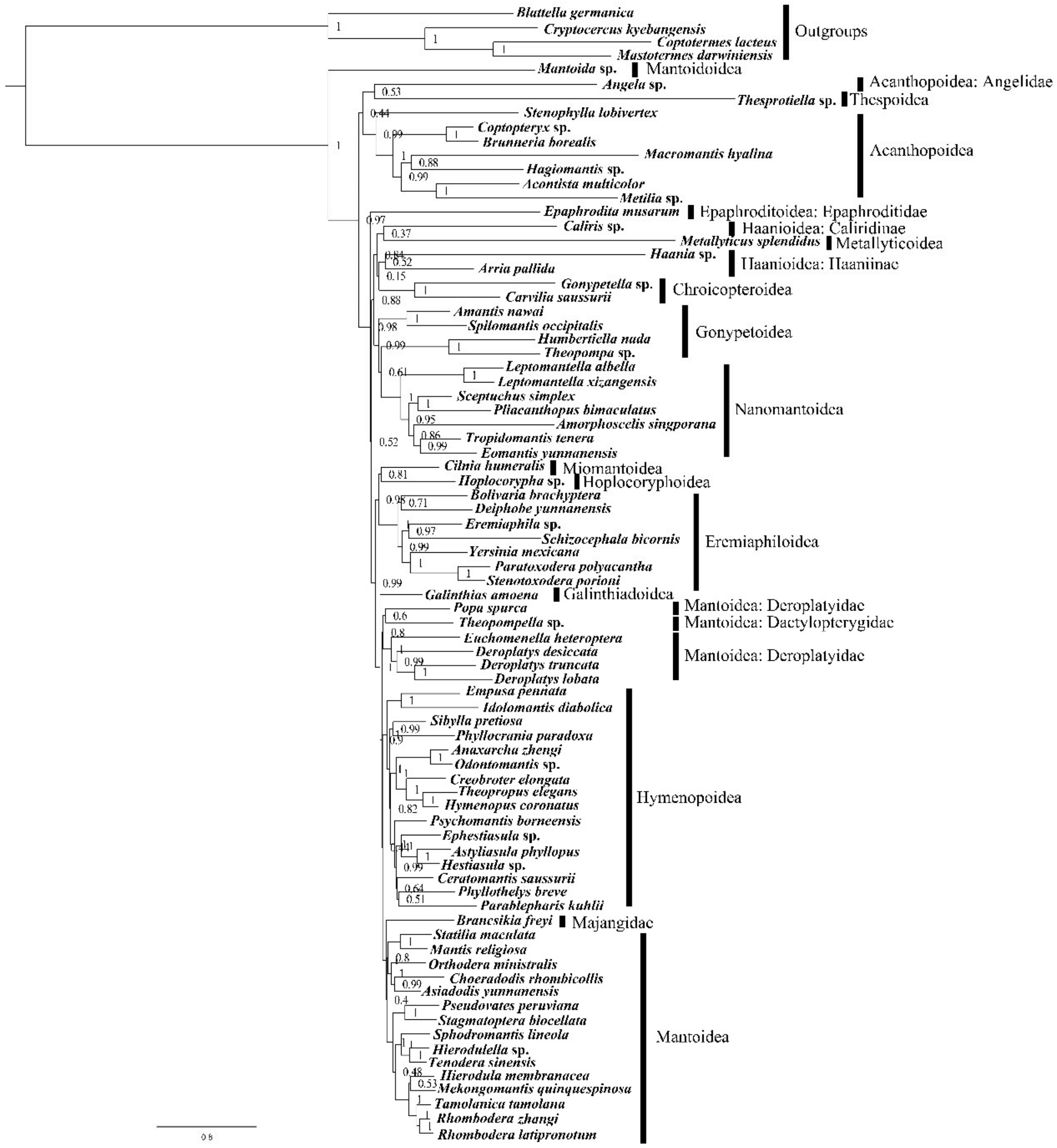

Figures S19. Phylogenetic tree inferred from BI analysis of NGPCGRNA using the site-heterogeneous model. Bayesian posterior probabilities (BPPs) of BI analyses were labeled at each node.

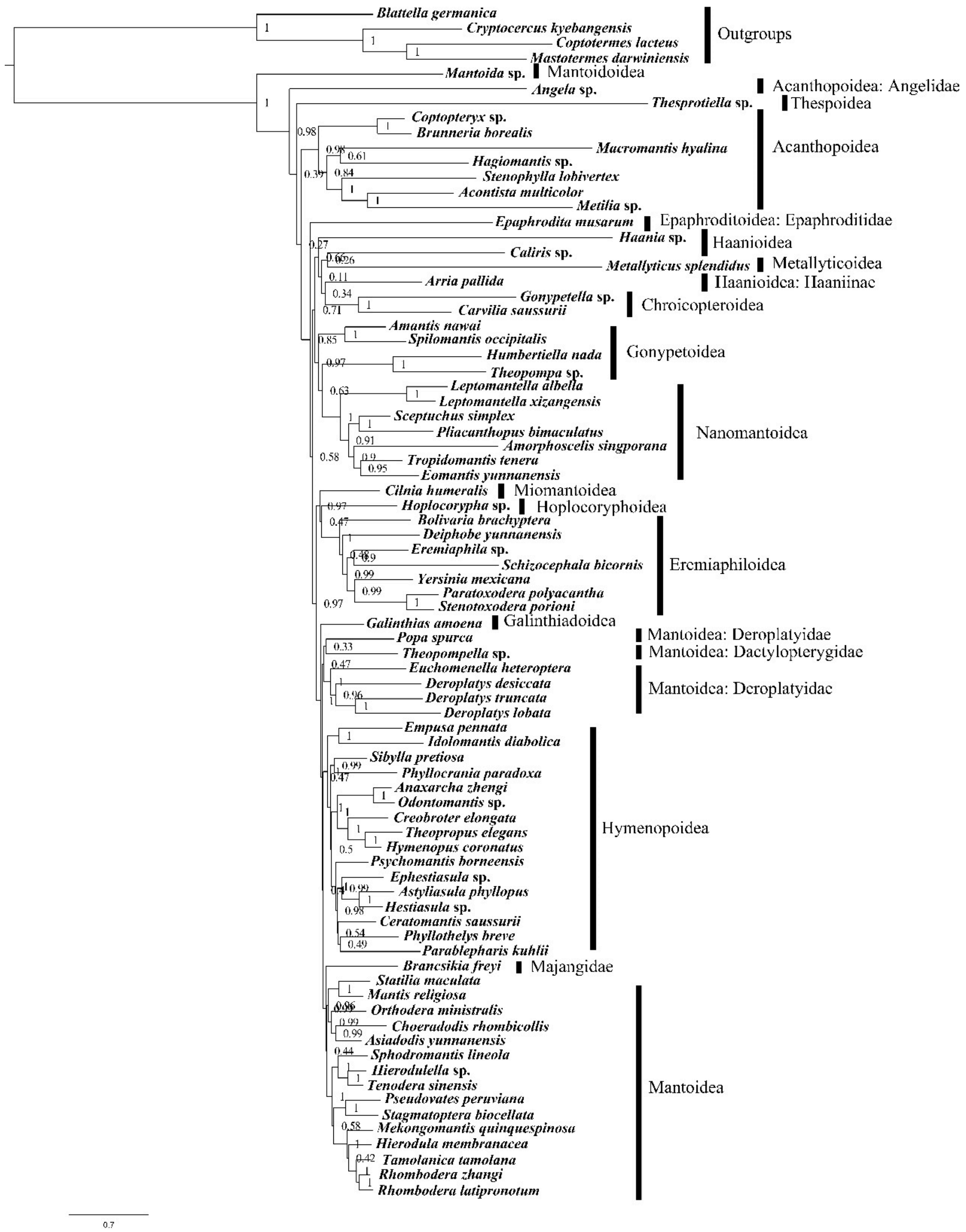

Figures S20. Phylogenetic tree inferred from BI analysis of PCGRNA using the site-heterogeneous model. BPPs of BI analyses were labeled at each node.

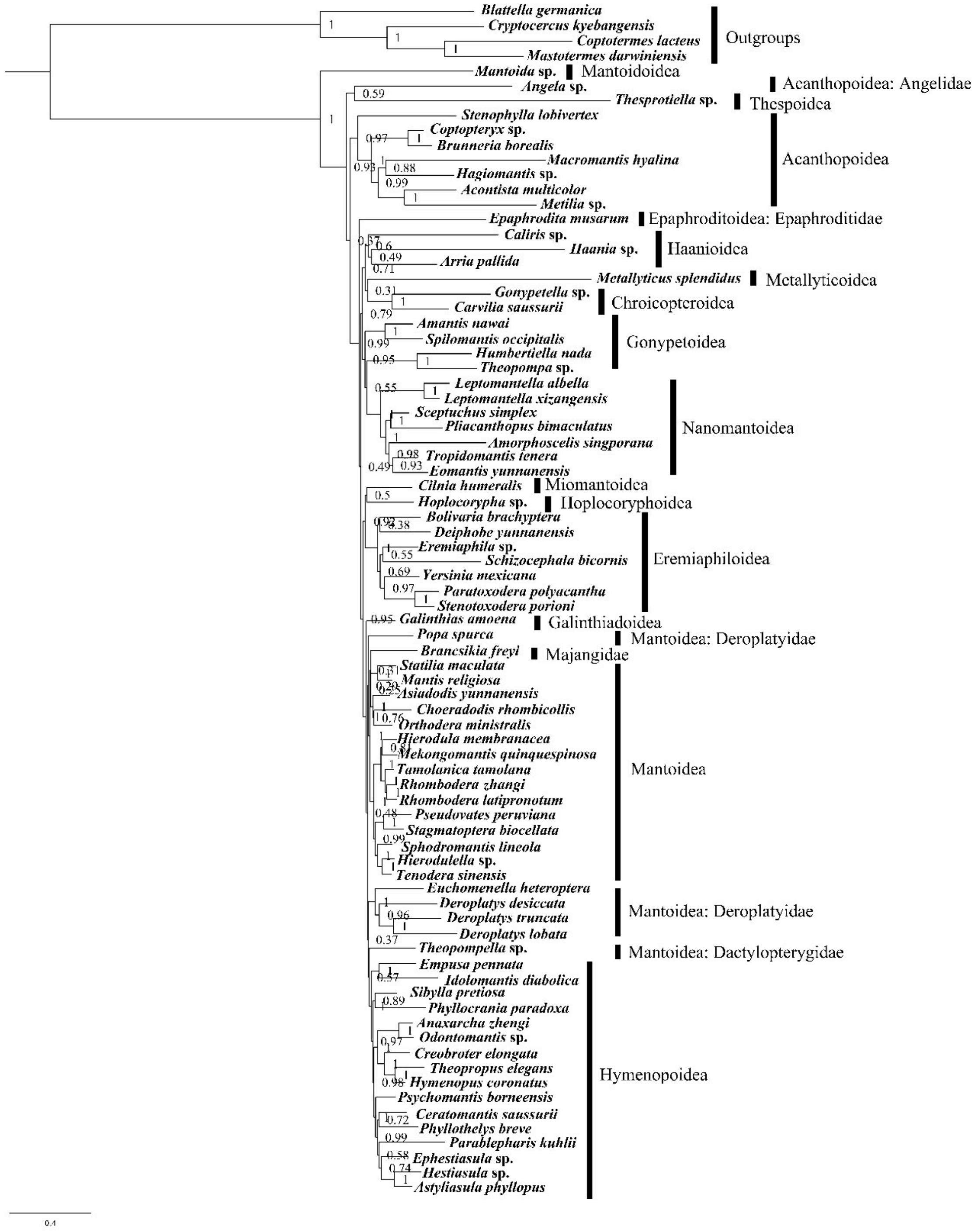

Figures S21. Phylogenetic tree inferred from BI analysis of NGPCG12RNA using the site-heterogeneous model. BPPs of BI analyses were labeled at each node.

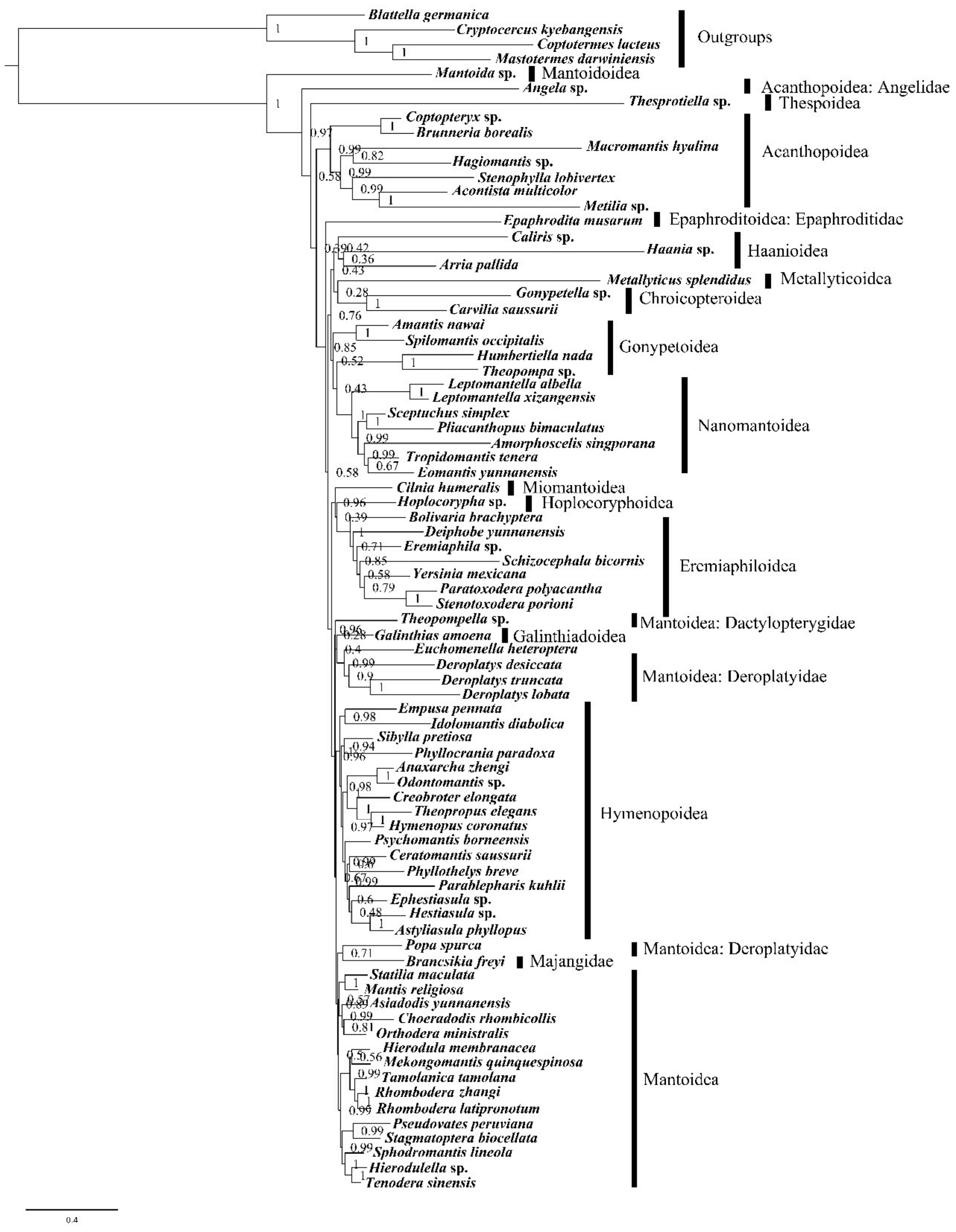

Figures S22. Phylogenetic tree inferred from BI analysis of PCG12RNA using the site-heterogeneous model. BPPs of BI analyses were labeled at each node.
